# Development and Optimization of Ionic Strength-Responsive Lipid–Polymer Hybrid Nanoparticles for Buccal Protein Delivery

**DOI:** 10.3390/pharmaceutics18060719

**Published:** 2026-06-11

**Authors:** Eslam Ramadan, Nooh Mdrmah, Martin Deák, Norbert Varga, Edit Csapó, Tamás Sovány, Katalin Kristó

**Affiliations:** 1 Institute of Pharmaceutical Technology and Regulatory Affairs, University of Szeged, Eötvös u. 6., H-6720 Szeged, Hungary; eslam.ramdan@mu.edu.eg (E.R.); deak.martin@szte.hu (M.D.); kristo.katalin@szte.hu (K.K.); 2 Department of Pharmaceutics, Faculty of Pharmacy, Minia University, Minia 61519, Egypt; 3 Graduate School of Health and Drug Sciences, University of Paris-Saclay, 17, Avenue des Sciences, 91400 Orsay, France; nooh.karama@gmail.com; 4 Interdisciplinary Excellence Center, Department of Physical Chemistry and Materials Science, University of Szeged, Rerrich B. Sqr. 1, H-6720 Szeged, Hungary; vargano@chem.u-szeged.hu (N.V.); juhaszne@chem.u-szeged.hu (E.C.); 5 MTA-SZTE Lendület “Momentum” Noble Metal Nanostructures Research Group, University of Szeged, Rerrich B. Sqr. 1, H-6720 Szeged, Hungary

**Keywords:** buccal, ethanol injection, ionic strength, LPHN, protein delivery, stimuli-responsive

## Abstract

**Background:** Oral protein delivery is a major challenge in the field of pharmaceutical technology due to poor stability and limited permeability through intestinal barriers. Buccal delivery is a promising alternative with less restricting physiological conditions; however, low protein permeability is still a limiting factor. Multiple nanocarriers have been proposed to improve buccal protein delivery with lipid–polymer hybrid nanoparticles (LPHNs) combining the advantages of both polymeric and lipid-based systems. However, these conventional carriers rely on passive protein protection and lack adaptive release mechanisms. **Objectives:** This work aimed to develop and systematically optimize an ionic strength-responsive LPHN system that can minimize protein release in buccal ionic conditions while offering a triggered release in plasma after absorption. **Methods:** LPHNs were prepared by a two-step approach where polymeric cores of Eudragit-L100 were prepared by electrostatic complexation with Lysozyme (LYZ) followed by lipid shell formation by the ethanol injection method. Systematic optimization was performed using two-level factorial and central composite designs. Moreover, the ionic strength responsiveness and in vitro LYZ release were investigated in different ionic strength media. **Results:** The final optimized formulations, LPHNs and sodium deoxycholate-containing LPHNs (NaDC-LPHNs), exhibited a particle size of 257.2 ± 1.5 nm and 246 ± 5.7 nm, encapsulation efficiency of 69.89 ± 0.22% and 68.14 ± 0.16%, and high drug loading efficiency of 24.11 ± 0.06% and 23.65 ± 0.04%, respectively. Moreover, both formulations showed minimal protein release at low ionic strength (buccal-like) conditions while demonstrating a triggered release at higher ionic strength (plasma-like) conditions. **Conclusions:** The developed system may provide a promising smart strategy to improve buccal protein delivery by enhancing buccal protection and improving systemic delivery.

## 1. Introduction

Oral protein delivery is extremely challenging due to multiple biological barriers throughout the gastrointestinal (GI) tract as well as the physicochemical properties of the drug itself. Biological barriers include protein denaturation in strong acidic conditions of the stomach, degradation by the abundant proteolytic enzymes in the intestine, low permeability for hydrophilic macromolecular drugs, and inactivation by the metabolic enzymes [1,2]. In addition, other drug-related factors such as low lipophilicity, large molecular size, and molecular complexity of peptides and proteins can pose additional challenges to their oral delivery. Together, these barriers result in very low protein stability and systemic bioavailability, restricting their oral administration in clinical settings [1,3].

Alternatively, the buccal route may provide a promising option for oral protein delivery, with nearly neutral pH conditions and low enzymatic activity compared to stomach and intestine, and avoidance of hepatic first-pass metabolism. Moreover, excellent accessibility, convenience, and ease of self-administration make the buccal route an attractive alternative to the invasive parenteral routes, enhancing patient compliance and adherence to the treatment [4]. Despite these advantages, buccal mucosa still has low permeability for hydrophilic protein- and peptide-type drugs. Therefore, multiple approaches have been explored to improve the buccal delivery of such macromolecules, including the use of penetration enhancers, chemical modification of proteins and peptides, and encapsulation within nanoparticulate delivery systems [4,5].

Several nanocarrier delivery systems, including liposomes, solid lipid nanoparticles (SLNs), nanostructured lipid carriers (NLCs), polymeric nanoparticles, and lipid–polymer hybrid nanoparticles (LPHNs) have been examined to improve stability and permeability of peptides and proteins after oral administration [6,7,8,9,10]. Amongst these nanocarriers, LPHNs emerged as a smart approach that combines the structural stability of polymeric nanocarriers with the biocompatibility of the lipid-based systems, offering improved protection and enhanced transmembrane permeability of therapeutic proteins and peptides [11]. These hybrid systems are formed of a core–shell structure with either lipid core–polymer shell or most commonly, polymer core–lipid shell. Encapsulating proteins and peptides in LPHNs can improve their stability and control drug release compared to lipid-based systems and provide better membrane interaction and transcellular absorption compared to polymeric nanocarriers [12]. These nanocarrier systems can improve permeability and oral absorption of the encapsulated protein therapeutics by helping penetrate the dense mucous layer and cross the epithelial barrier. Physicochemical properties like particle size, surface chemistry, and surface charge of nanocarriers are the main factors controlling their mucous penetration and epithelial absorption. For example, nanoparticles sized roughly between 100 and 300 nm have been reported for buccal or sublingual drug delivery, with the 200 nm nanoparticles penetrating deeper and faster through the buccal mucosa [13,14]. Likewise, negatively charged nanoparticles and particles with hydrophilic surfaces showed better mucous penetration capability compared to positively charged and hydrophobic ones [11,13]. After crossing the mucous layer, these nanocarriers come into contact with the absorptive epithelial layer where they can be transported through paracellular, transcellular, receptor-mediated, or lymphatic transport, improving the delivery efficiency of the encapsulated protein drugs [11].

**Figure 1:**
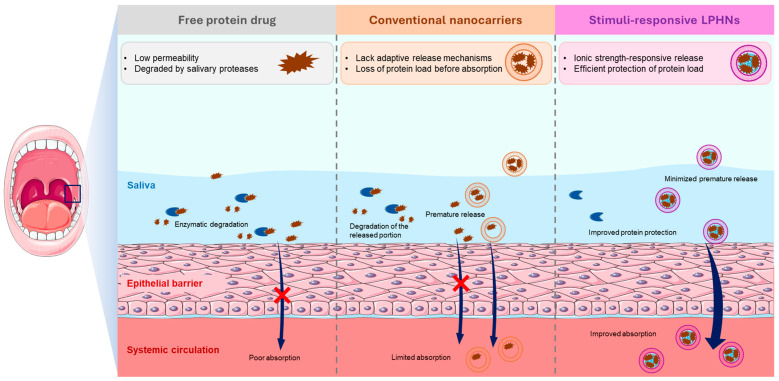
Schematic illustration showing challenges of buccal protein delivery and the potential advantages of ionic strength-responsive nanocarriers.

However, these conventional nanocarriers often provide passive payload protection and lack adaptive release mechanisms, leading to significant loss of encapsulated drugs before being absorbed and transferred to the site of action (Figure 1).

Stimuli-responsive nanocarriers have emerged as advanced generations of drug delivery systems that allow a site-specific and on-demand drug release, providing maximum drug protection and higher delivery efficiency [15,16]. These systems are designed to release their encapsulated payloads in response to multiple physiological or external triggers such as pH, temperature, redox conditions, enzymes, ultrasound, and magnetic fields [17,18,19,20,21,22]. In the context of oral protein delivery, these smart systems are particularly advantageous as they can provide higher protection of these delicate therapeutics and minimize their loss prior to systemic absorption, improving their overall bioavailability and therapeutic efficacy [15]. Yet stimuli-responsive systems specifically tailored for the buccal protein delivery remain insufficiently explored. Therefore, our goal was to develop and systematically optimize a stimuli-responsive LPHN delivery system for buccal protein delivery.

Ionic strength as a stimulus for triggered protein release has been reported in the literature since the late 1990s. Early work on pH- and ionic strength-responsive hydrogel and microgel formulations showed that changes in ionic strength can modulate protein release from the gel matrix by deswelling or collapse of the polymer network caused by disturbance of electrostatic interactions between the polymer components and/or the entrapped protein [23,24]. However, despite the early efforts, application of such principles to nanocarrier protein delivery systems is still an undiscovered area of research.

The buccal cavity has a distinct physiological environment, with watery salivary secretions that have low electrolyte concentrations compared to human plasma. Human salivary secretions were reported to contain Na^+^, K^+^, Cl^−^, and HCO_3_^−^ in a concentration of 3.6–20.8, 13.7–30.7, 15.8–38.8, and 6.4–58.8 mM, respectively, with the lower limits being observed at resting conditions, corresponding to a total ionic strength of ~30–40 mM [25]. This value is very low compared to the plasma ionic strength, which is typically ~150 mM due to the high concentrations of Na^+^ and Cl^−^ ions (~140 and ~105 mM, respectively) [26]. This wide difference in ionic strength between buccal and plasma conditions allowed us to design a novel ionic strength-responsive LPHN system, which maintains low protein release at the low ionic strength of saliva, while enabling a triggered release at high ionic strength plasma conditions after nanoparticle absorption. To the best of our knowledge, this is the first ionic strength-responsive nanoparticle delivery system to be designed for buccal protein delivery, with the aim of improving delivery efficiency and bioavailability by both protein protection in pre-absorption settings and permeability enhancement using phospholipids and penetration enhancers.

In this work, lysozyme (LYZ) was used as a model protein drug due to its wide-range antimicrobial, antiviral and immunomodulatory activity, structural complexity, and its ability to form ionic complexes with negatively charged polymers [27]. Eudragit L-100 (Eud-L100) copolymer was used as the polymer core material for its anionic nature that allows efficient complexation with LYZ with high encapsulation efficiency (EE). This electrostatic complexation between LYZ and Eud-L100 polymer is the basis for the ionic strength responsiveness of the developed LPHN system, utilizing the charge screening principle. Furthermore, a lipid shell of Soy L-α-phosphatidylcholine (PC), a natural phospholipid known for its safety and biocompatibility, was formed around the polymeric cores using the ethanol injection method. In addition, sodium deoxycholate (NaDC), a known penetration enhancer, was incorporated in the final optimized LPHN formulation to provide further enhancement in nanoparticle permeability through buccal mucosa, eventually improving bioavailability. The factors affecting physicochemical properties of the formed nanoparticles in both polymer core and lipid shell formation steps were investigated and systematically optimized using appropriate experimental designs. Finally, the optimized formulations were investigated for their ionic strength responsiveness and LYZ release kinetics in different ionic conditions resembling both buccal and plasma environments.

## 2. Materials and Methods

### 2.1. Materials

Eudragit^®^ L100 (Eud-L100) (methacrylic acid-methyl methacrylate copolymer 1:1) and lyophilized powder of lysozyme (LYZ) from chicken egg white were obtained from Evonik Röhm GmbH (Darmstadt, Germany) and MedChemExpress (Monmouth, NJ, USA) respectively. Soy L-α-phosphatidylcholine (95%) (PC) was purchased from Avanti Research (Alabaster, AL, USA). Sodium deoxycholate (NaDC) was purchased from Sigma-Aldrich^®^ (St. Louis, MO, USA). Tween 80^®^ (polysorbate 80) was purchased from Merck KGaA (Darmstadt, Germany). Other materials were of analytical grade and purified water was used for preparation of different solutions and buffers.

### 2.2. Preparation of Eud-LYZ Polymeric Core

**Figure 2:**
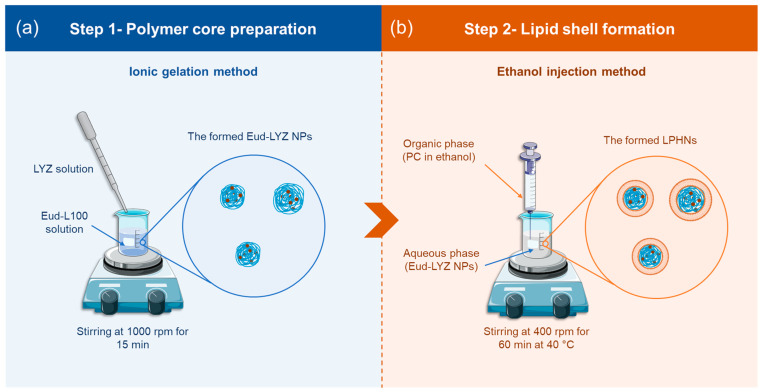
Schematic illustration of LPHN preparation process. (**a**) shows polymer core preparation step, while (**b**) demonstrates the ethanol injection setting of lipid shell formation step.

LPHNs were prepared by the two-step approach, where polymeric core and lipid shell formation were carried out in two independent processes. Polymeric cores were prepared by the ionic gelation method where the positively charged LYZ is complexed with the negatively charged Eud-L100 molecules by electrostatic interactions. First, stock solutions of Eud-L100 (3 mg/mL) and LYZ (3 mg/mL) were prepared in PBS buffers of different pH values (6.8, 7.8, and 8.8). Both solutions were filtered through 0.4 µm hydrophilic PTFE membranes to remove undissolved material and aggregates, then diluted with the same buffer to achieve the required Eud-L100 concentration (0.5, 1, or 1.5 mg/mL) and Eud/LYZ ratio (1, 1.5, or 2 *w*/*w*) in the final polymeric nanoparticles. For each formulation, 5 mL of LYZ solution was added dropwise to 5 mL of Eud-L100 solution on a magnetic stirrer under constant stirring at 1000 rpm and 25, 37.5 or 50 °C as indicated by the experimental design. The mixtures were stirred for additional 15 min to allow for complete interaction and nanoparticle formation (Figure 2a). Finally, the prepared nanoparticle dispersions were stored in the refrigerator at 2–8 °C until physicochemical characterization.

### 2.3. Preparation of LPHNs

The optimized formulation of Eud-LYZ nanoparticles was used for the preparation of the LPHNs using the ethanol injection method [28]. The organic phase was prepared by dissolving PC in ethanol (96%) (20 mg/mL), while an Eud-LYZ nanoparticle dispersion containing Tween 80 (0.2 *w*/*w* of the lipid concentration) was used as the aqueous phase. For each preparation, the PC solution was diluted with ethanol to give the required Lipid/Polymer mass ratio and Aqueous/Organic volume ratio. The required volume of the organic phase was transferred to a 3 mL syringe with a 23 G needle and allowed to flow (~0.5 mL/min) into a beaker containing 10 mL of the aqueous phase under continuous stirring at 400 rpm and 40 °C (Figure 2b). The formed LPHNs were stirred for additional 1 h to allow for nanoparticle assembly and complete ethanol evaporation.

For preparation of NaDC-containing LPHNs, the same steps were followed except that a volume of NaDC solution (20 mg/mL in ethanol) equivalent to 0.2 *w*/*w* of the PC concentration was added to the organic phase during the dilution step. LPHNs formation was indicated by the milky turbid appearance formed in the aqueous phase while the organic phase was injected. The prepared LPHN formulations were stored at 2–8 °C until characterization and further experimentation.

### 2.4. Characterization of LPHNs

#### 2.4.1. Determination of Particle Size, Polydispersity Index, and Zeta Potential

Particle size, polydispersity index (PDI), and zeta potential (ZP) of the prepared Eud-LYZ nanoparticles and LPHNs were measured by dynamic and electrophoretic light scattering using HORIBA nanoPartica SZ-100 Nanoparticle Analyzer (Kyoto, Japan). Aliquots of 200 µL were diluted to 2 mL with distilled water and moved to a specific cuvette for either particle size and PDI analysis or ZP measurement. All measurements were done in triplicate.

#### 2.4.2. Determination of LYZ Encapsulation and Drug Loading Efficiencies

Encapsulation efficiency (EE) and drug loading efficiency (DLE) of LYZ in the prepared NPs were determined by the indirect method. Briefly, 2 mL of the NP dispersions were centrifuged for 30 min at 16,500 rpm (~25,900× *g*) and 4 °C to separate the unentrapped LYZ. The concentration of free LYZ in the supernatant was measured by UV-VIS spectrophotometry at 281 nm and calculated based on a calibration curve constructed using different known LYZ concentrations in the same buffer used for NP preparation. The encapsulated LYZ concentration was calculated by subtracting the free LYZ concentration from the total initial concentration then encapsulation and loading efficiencies were calculated according to the following equations (Equations (1) and (2)):

(1) $$EE\%=\frac{{LYZ}_{o}-{LYZ}_{s}}{{LYZ}_{o}}\times 100$$

(2) $$DLE\%=\frac{{LYZ}_{o}-{LYZ}_{s}}{\left({LYZ}_{o}-{LYZ}_{s}\right)+Eud+L+T+DC}\times 100$$

where ${LYZ}_{o}$ is the initial LYZ concentration, ${LYZ}_{s}$ is LYZ concentration in supernatant, Eud is the polymer concentration, L is the lipid concentration, T is the tween 80 concentration, and DC is the concentration of NaDC in the final LPHNs.

### 2.5. Optimization of LPHN Preparation Steps

For optimization of the polymeric Eud-LYZ NPs, a half-replicate two-level factorial design with four factors (2^4^) was generated using Design-Expert^®^ software, version 13.0.5.0 (Stat-Ease Inc., Minneapolis, MN, USA). The design, which had 8 formulations, representing different factor combinations, plus one center point for curvature estimation, enables the reduction of the number of experiments but it should be noted that in this case main factor effects and 2-way interactions may be confounded with higher order interactions. Eud-L100 concentration (A), Eud/LYZ ratio (B), buffer pH (C), and mixing temperature (D) were selected as the independent variables (factors), while particle size, PDI, ZP, and LYZ EE were chosen as the dependent variables (responses) (Table 1). Lower and upper limits of Eud-L100 concentration and Eud/LYZ ratio were selected based on literature data and preliminary LYZ solubility observations [29]. Visible aggregations were observed in LYZ solutions above 3 mg/mL in different PBS buffers (pH 6.8, 7.8, and 8.8), while 1.5 mg/mL LYZ concentration showed practically acceptable appearance with minimal aggregations (Figure S1). Therefore, the limits of Eud/LYZ ratio and Eud-L100 concentration were selected to maintain a LYZ concentration less than 1.5 mg/mL in the final formulation to avoid formation of large aggregates or interference with particle size measurements. The rationale behind the limits of the other factors is presented in Table 1.

After preparation, the responses were measured and the collected data were statistically analyzed using the software and fitted to the appropriate model. The model fit and prediction accuracy were assessed using R-squared value (R^2^) and the scatterplot of actual versus predicted values. Finally, the 3D surface graphs were generated using the software to describe the effects of different factors on each response.

On the other hand, optimization of the ethanol injection method for LPHN preparation was achieved by a central composite design with two central points. Lipid/Polymer (L/P) mass ratio (E) and Aqueous/Organic (A/O) volume ratio (F) were selected as independent variables, while particle size, PDI, ZP, LYZ EE, and DLE were used as responses, in addition to a binary response introduced later to visualize the formulation failure zone in the design space (Table 2). Statistical analysis and model fitting of the acquired experimental data were carried out and response surface graphs were generated to visualize the effects of different factors on each response. Experimental data were statistically analyzed using an ANOVA test to identify significant model terms and overall model significance. Model fit statistics were evaluated using R^2^, adjusted R^2^, and predicted R^2^. Terms with *p* < 0.05 were considered statistically significant.

For both designs, numerical and graphical optimization functions were used to select the optimal formulations based on the desired response goals, and to set the boundaries for the working design space. In case of Eud-LYZ NPs, the goals were set to minimize particle size and PDI and maximize EE%. Likewise, the goals for optimal LPHNs were the same as Eud-LYZ NPs, in addition to minimizing the formulation failure and maximizing the DLE. The predicted optimized formulations of Eud-LYZ NPs and LPHNs were prepared in triplicate, and the obtained experimental data were compared to the predicted responses to confirm and validate the optimization process.

### 2.6. Effect of Ionic Strength on LYZ Release Behavior

The effect of ionic strength on LYZ release from Eud-LYZ NPs, LPHNs, and NaDC-LPHNs was investigated using buffer media (pH 6.8) of varying ionic strengths. Aliquots (0.25 mL) of each formulation were transferred into 2 mL Eppendorf tubes and diluted to a final volume of 1.5 mL using buffer solutions adjusted to ionic strengths of 30, 50, 100, 150, and 200 mM using NaCl. The samples were incubated in a water bath for 30 min at 37 °C to allow ionic strength–induced LYZ release. Following incubation, the samples were centrifuged as mentioned previously to separate the released (free) LYZ from the nanoparticulate fraction. The concentration of LYZ in the supernatant was quantified by UV-VIS spectrophotometry at 281 nm using an appropriate calibration curve.

To account for unentrapped (free) LYZ present prior to the release experiment, the initial free LYZ content (time zero) was determined separately. Briefly, 0.25 mL of each formulation was diluted to 1.5 mL with PBS (pH 6.8 and 10 mM ionic strength), followed by immediate centrifugation. The LYZ concentration in the resulting supernatant was measured spectrophotometrically and considered as the unencapsulated fraction. The amount of LYZ released from the encapsulated fraction was calculated by subtracting the initial free LYZ concentration (time zero) from the total LYZ concentration measured after 30 min incubation at each ionic strength. All experiments were performed in triplicate under identical conditions to ensure reproducibility.

### 2.7. In-Vitro LYZ Release

In vitro LYZ release from different nanoparticle formulations was performed using sample-and-separate method as previously reported, with some modifications [34]. Briefly, 0.75 mL of each formulation was diluted to 1 mL with PBS (pH 6.8 and 10 mM buffer strength) and immediately centrifuged to determine the initial LYZ concentration at time zero. After removing free LYZ, the pellets were resuspended in 1 mL of the release medium and incubated in water bath at 37 °C. At different time points (30, 60, 120, 180, 300, and 420 min), samples were centrifuged for 30 min at 16,500 rpm (~25,900× *g*) and 4 °C to separate the released LYZ in the supernatant, and pellets were resuspended in 1 mL of fresh release medium. The test was carried out in two stages: The first 60 min was performed in PBS with a pH of 6.8 and ionic strength of 30 mM to mimic the ionic strength of human saliva in pre-absorption stage. After 60 min, the release medium was replaced with a plasma-resembling PBS with a pH of 7.4 and ionic strength of 150 mM to mimic the post-absorption conditions. All experiments were performed in triplicate and data were reported as mean ± SD.

### 2.8. Storage Stability Test

Stability of LPHNs and NaDC-LPHNs was studied over a one month storage period. Samples were prepared as previously mentioned and divided into two portions to be stored in two different conditions: at ambient room temperature (~25 °C) and in refrigerator (~4 °C). At predetermined time points (0, 7, 14, 21, and 28 days), samples were taken for particle size, PDI, ZP, and EE measurements. All the measurements were performed in triplicate and data were presented as mean ± SD.

### 2.9. Fourier Transform Infrared (FT-IR) Spectroscopy

The interaction between LYZ and other formulation components was examined using Avatar 330 Thermo Nicolet FT-IR spectrometer running on OMNIC software, version 6.1a (Thermo Fisher Scientific Inc., Waltham, MA, USA). Samples from LPHNs and NaDC-LPHNs were examined using potassium bromide (KBr) disk method. Briefly, small amounts of freeze-dried samples (2–3 mg) were mixed with 0.2 g of KBr, palletized under vacuum, and measured across a range of wavenumber from 4000 to 400 cm^−1^. For data acquisition, spectra were obtained from 128 scans with 4 cm^−1^ optical resolution using OMNIC software. Finally, SpectraGryph software (version 1.2.15., F. Menges Software Entwicklung, Germany) was used for spectral analysis.

### 2.10. Statistical Analysis

The statistical analysis on the effect of ionic strength and NP type on the release characteristics of LYZ was performed with Statistica software v.14.1.0.25 (TIBCO Software Inc., Palo Alto, CA, USA) using factorial ANOVA and Bonferroni’s post hoc comparison. Likewise, statistical significance of the effects of time and NP type on particle size, PDI, ZP, and EE during storage at 4 and 25 °C were examined following the same test.

## 3. Results and Discussion

### 3.1. Polymer Core Optimization

#### 3.1.1. Effects of Independent Variables on the Particle Size and PDI of Eud-LYZ NPs

The particle size and PDI values of the prepared Eud-LYZ NPs ranged from 217.3 to 526 nm and from 0.061 to 1.299 respectively for different combinations in the design matrix (Table 3).

The data showed good model fitting to the selected factorial model with R^2^ of 1, adjusted R^2^ of 0.9999, and predicted R^2^ of 0.9898 for response particle size, and R^2^ of 0.9998, adjusted R^2^ of 0.9980, and predicted R^2^ of 0.8593 for response PDI. ANOVA results revealed that pH, temperature, and Eud/LYZ ratio had significant effects on the particle size of Eud-LYZ NPs, while the effect of Eud-L100 concentration was insignificant. On the other hand, the response PDI was significantly affected by all the factors studied. The factor coefficients for the particle size and PDI responses are summarized in the following equations (Equations (3) and (4)):

(3) Size=317.69+1.18A +16.10B+77.14C−46.01D−39.19AB+2.55AC+33.75AD 

(4) PDI=0.3373−0.1615A+0.1090B+0.1775C−0.1621D−0.0702AB−0.1696AC+0.1097AD

where (*A*) is Eud-L100 concentration, (*B*) is Eud/LYZ ratio, (*C*) is pH, and (*D*) is the temperature.

**Figure 3:**
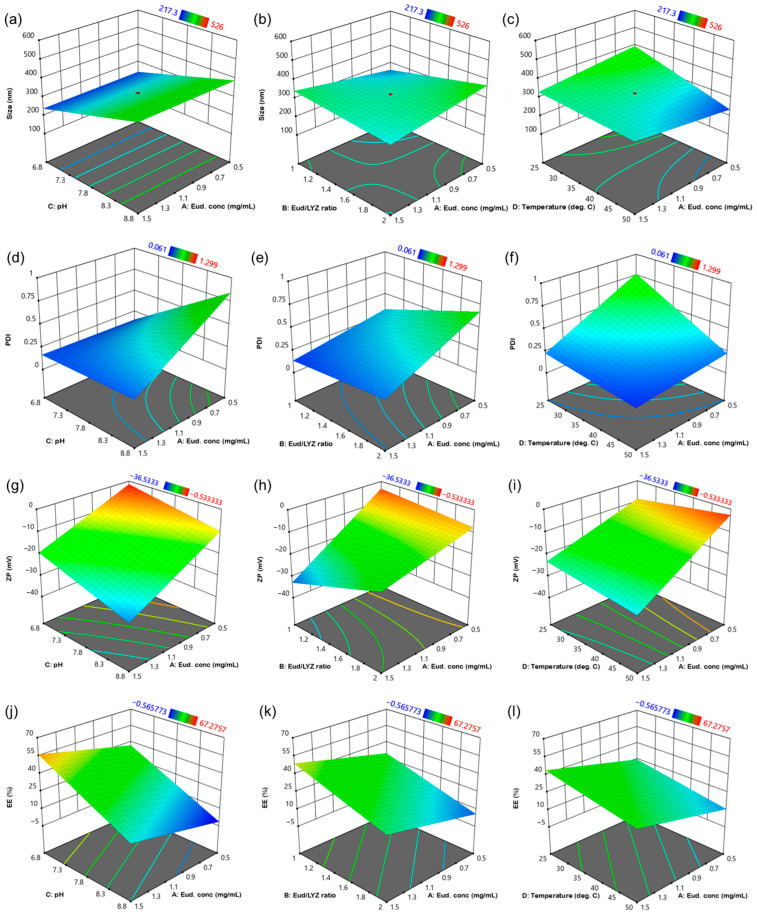
3D surface plots of different responses. The figure shows the effects of Eud-L100 concentration vs. pH (**a**), Eud/LYZ ratio (**b**), and temperature (**c**) on particle size; pH (**d**), Eud/LYZ ratio (**e**), and temperature (**f**) on PDI; pH (**g**), Eud/LYZ ratio (**h**), and temperature (**i**) on ZP; and pH (**j**), Eud/LYZ ratio (**k**), and temperature (**l**) on EE of the polymeric Eud-LYZ NPs. Each plot represents the effects of two factors on one response while maintaining the other two factors fixed at their center levels.

Decreasing pH from 8.8 to 6.8 led to a significant reduction in particle size and PDI from around 400 nm to less than 250 nm, and from more than 0.8 to less than 0.2, respectively (Figure 3a,d). This effect can be attributed to the change in LYZ net charge at different pH conditions. Generally, at low pH values, more of basic amino acids of LYZ become protonated, and the overall net charge increases, which allows stronger interaction with the negatively charged Eud-L100 polymer and formation of compact, small and uniform Eud-LYZ NPs [31].

The change in Eud/LYZ ratio and temperature exerts more considerable effect at low Eud-L100 concentration where decreasing Eud/LYZ ratio from 2 *w*/*w* to 1 *w*/*w* or increasing temperature from 25 °C to 50 °C led to the significant reduction in particle size and PDI. While at high Eud-L100 concentration, both factors showed minimal effects (Figure 3b,c,e,f). The effect of temperature is possibly attributed to the reduced solution viscosity and increased molecular motions due to the increased kinetic energy, which accelerate polymer-protein interactions and shorten particle growth time. This leads to the formation of smaller and more uniform particles compared to lower temperatures. This result is consistent with a previous study by Jones and McClements (2010), where they reported a similar temperature-dependent particle size reduction in β-lactoglobulin-pectin complexes from 310 to 280 nm when temperature increased from 70 to 90 °C [35]. On the other hand, a high Eud/LYZ ratio introduces excess polymer, leading to increased polymer-polymer repulsions at the polymer-rich surface, which in turn increases the apparent particle size and PDI measured by DLS.

#### 3.1.2. Effects of Independent Variables on the ZP of Eud-LYZ NPs

The measured ZP values of different design formulations ranged from −36.53 to −0.53 mV. Model fit statistics of these data showed good model fitting with high R^2^, adjusted R^2^, and predicted R^2^ values of 1, 0.9999, and 0.9947 respectively. ANOVA results showed that Eud-L100 concentration, pH, and Eud/LYZ ratio had significant effects on ZP, while the effect of temperature was insignificant. These effects are summarized in the response equation (Equation (5)). Increasing Eud-L100 concentration resulted in a progressive reduction in ZP of the nanoparticles (Figure 3g–i), likely due to the formation of a denser outer polymer layer composed of the negatively charged Eud-L100 at higher polymer levels. In contrast, at lower Eud-L100 concentrations, the polymer layer becomes more diffuse and highly hydrated, leading to a decreased surface density of negative charges on the nanoparticle interface, manifested as increase in the measured ZP.

(5) ZP=−15.50−9.96A+2.58B−5.12C+0.3417D+4.74AB−1.04AC−2.73AD

where (*A*) is Eud-L100 concentration, (*B*) is Eud/LYZ ratio, (*C*) is pH, and (*D*) is the temperature.

ZP was also dependent on the pH where increasing pH from 6.8 to 8.8 led to a significant reduction in the measured ZP values (Figure 3g). This behavior is consistent with the pH-responsive ionization characteristics of both formulation components: LYZ and Eud-L100. At lower pH values, LYZ carries a substantially higher number of positively charged groups (nearly +8 at pH 6.8), whereas its protonation decreases markedly at elevated pH (net charged groups around +1.5 at pH 8.8) [31,36]. In parallel, Eud-L100 is only partially ionized at pH 6.8, exhibiting a moderately negative charge, but becomes fully ionized at pH 8.8, resulting in a strong anionic polymer [37]. The combined ionization behavior of both components at lower pH promotes the formation of tightly bound, electrostatically stabilized complexes, yielding nanoparticles with relatively higher surface potential (less negative ZP) due to the strong contribution of the highly cationic LYZ. In contrast, at higher pH, the reduced positive charge on LYZ shifts the primary charge contribution to the fully ionized, negatively charged polymer, leading to nanoparticles with increasingly negative surface charges.

The influence of Eud/LYZ ratio on ZP became apparent only at high Eud-L100 concentrations. Unexpectedly, increasing Eud/LYZ ratio resulted in a less negative ZP, despite the anticipated increase in negative charge associated with higher polymer content (Figure 3h). This counterintuitive trend can be attributed to structural differences on the nanoparticle surface. At high Eud-L100 concentrations, lower Eud/LYZ ratios of 1/1 *w*/*w* (high LYZ concentration) promote stronger electrostatic interactions between the polymer and LYZ, yielding more compact nanoparticles with a dense polymer layer at the surface that effectively shields the inner positive protein charges. Conversely, higher Eud/LYZ ratios of 2/1 *w*/*w* (lower LYZ concentration) reduce these electrostatic interactions, producing a more diffuse outer polymer layer with lower charge density, ultimately leading to a less negative measured surface potential.

#### 3.1.3. Effects of Independent Variables on LYZ Encapsulation Efficiency

The calculated EE data ranged from almost 0 to 67.28% for different design points. Model fitting of these data showed R^2^ value of 0.9114, adjusted R^2^ of 0.8229, and predicted R^2^ of 0.7344, indicating acceptable model fitting. Statistical ANOVA analysis revealed two significant factors: pH, and Eud/LYZ ratio, while the effects of temperature and Eud-L100 concentration were insignificant. The coefficients of these effects are shown in equation (Equation (6)). As expected, increasing pH from 6.8 to 8.8 significantly reduced LYZ encapsulation (Figure 3j) due to the previously discussed pH-dependent changes in the ionization status and the net charge value of LYZ, which promote stronger electrostatic attractions and consequently higher LYZ encapsulation at lower pH.

(6) EE=27.5023+9.55953A−11.9975B−19.2311C−7.03122D

where (*A*) is Eud-L100 concentration, (*B*) is Eud/LYZ ratio, (*C*) is pH, and (*D*) is the temperature.

For the Eud/LYZ ratio, we found that increasing the ratio from 1 to 2 led to a significant reduction in LYZ encapsulation, contrary to the expected enhancement associated with an excess of the oppositely charged polymer (Figure 3k). Although the exact cause of this effect is not clear, it might be attributed to the weaker interactions between LYZ and Eud-L100 polymer when the LYZ concentration is very low. At a low LYZ concentration, LYZ molecules may fail to form a particulate complex that can be precipitated upon centrifugation; instead, they can just be adsorbed to the surface of the soluble Eud-L100 molecules and still be detectable in the supernatant. Thus, a negligible EE can be detected at high Eud/LYZ ratios.

Moreover, increasing the Eud-L100 concentration or lowering the temperature led to a noticeable increase in LYZ encapsulation in Eud-LYZ polymeric NPs (Figure 3l); however, these effects were not statistically significant.

#### 3.1.4. Selection and Validation of an Optimized Eud-LYZ NP Formulation

**Figure 4:**
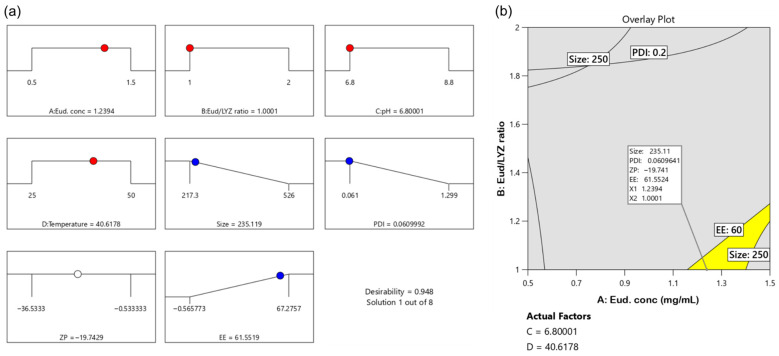
Numerical and graphical optimization of Eud-LYZ NPs. The figure shows the numerical optimization ramps representing the optimal factor conditions and the predicted responses (**a**), and the design space region (in yellow color) within the overall model space (**b**).

Numerical and graphical optimization functions were employed to identify the optimal formulation parameters and set the boundaries for the working design space. Numerical optimization criteria were set to minimize particle size and PDI while maximizing LYZ EE (Figure 4a). Meanwhile, design space boundaries were defined as the region combining particle size < 250 nm, PDI < 0.3, and EE > 60% as shown in Figure 4b. Among the nine candidate solutions generated by the software, the formulation exhibiting the highest overall desirability was selected as the optimized condition. This formulation comprised an Eud-L100 concentration of 1.24 mg/mL, an Eud/LYZ ratio of 1 *w*/*w*, a pH of 6.8, and a mixing temperature of 40.6 °C. Under these conditions, the model predicted a particle size of 235.1 nm, a PDI of 0.061, a ZP of −19.74 mV, and an EE of 61.55%.

To validate the optimization outcomes, three independently prepared batches of the optimized formulation were prepared and characterized. The experimentally obtained values closely matched the predicted responses, with all measured parameters falling within the respective 95% confidence intervals (Table 4). These findings confirm the robustness of the optimization approach and verify the successful development of the optimized Eud-LYZ nanoparticles.

### 3.2. Optimization of LPHNs

The ethanol injection method was employed to assemble a bilayer lipid shell around the optimized Eud–LYZ polymeric core. This technique, widely utilized in liposome preparation, relies on the spontaneous self-assembly of phospholipids upon their transition from an organic to an aqueous environment [28,38]. Its use enables precise control over nanoparticle size and polydispersity while maintaining a straightforward, resource-efficient workflow. Moreover, the method does not require specialized instrumentation, making it a labor- and cost-effective approach for the reproducible fabrication of LPHNs [39,40].

To optimize the ethanol injection method for LPHN preparation, an initial two-level factorial design with two center points was employed to evaluate the effects of the L/P weight ratio and A/O volume ratio on the physicochemical properties of the resulting nanoparticles. However, a significant curvature was observed in the particle size response, indicating non-linearity within the design space. Therefore, the experimental design was expanded into a response surface central composite design by incorporating additional runs to more comprehensively visualize the non-linear factor effects.

Originally, particle size, PDI, ZP, EE, and DLE were designated as the primary responses. However, one of the additional central composite design points exhibited a pronounced aggregation, yielding abnormally high particle size and PDI values. Consequently, a supplementary binary response was introduced to delineate the formulation failure region within the design space. The aggregated data point was then excluded from particle size and PDI analyses to prevent model distortion, as its inflated measurements reflected aggregate formation rather than the true nanoparticle dimensions.

#### 3.2.1. Effect of the Studied Factors on LPHNs’ Particle Size and PDI

LPHNs showed a marked increase in particle size relative to Eud-LYZ NPs, confirming the successful assembly of the lipid shell. The measured particle size and PDI values ranged from 217.8 to 357.3 nm and 0.150 to 0.371, respectively, excluding the aggregated formulation (Table 5). Statistical modeling demonstrated excellent fit with high R^2^, adjusted R^2^, and predicted R^2^ values of 0.9962, 0.9867, and 0.9199 for particle size, and 0.9932, 0.9760, and 0.8012 for PDI, respectively. ANOVA further indicated that particle size was significantly affected by both A/O ratio and L/P ratio, while PDI was only governed by the A/O ratio. As indicated by the polynomial response equations (Equations (7) and (8)), the A/O ratio showed more prominent effects on both particle size and PDI responses.

(7) $$Size=251.60-24.42E-42.60F+10.11EF+3.45E^{2}+18.91F^{2}$$

(8) $$PDI=0.2250+0.0044E-0.0836F-0.0069EF+0.0068E^{2}+0.0275F^{2}$$

where (*E*) Is L/P ratio, and (*F*) is A/O ratio.

**Figure 5:**
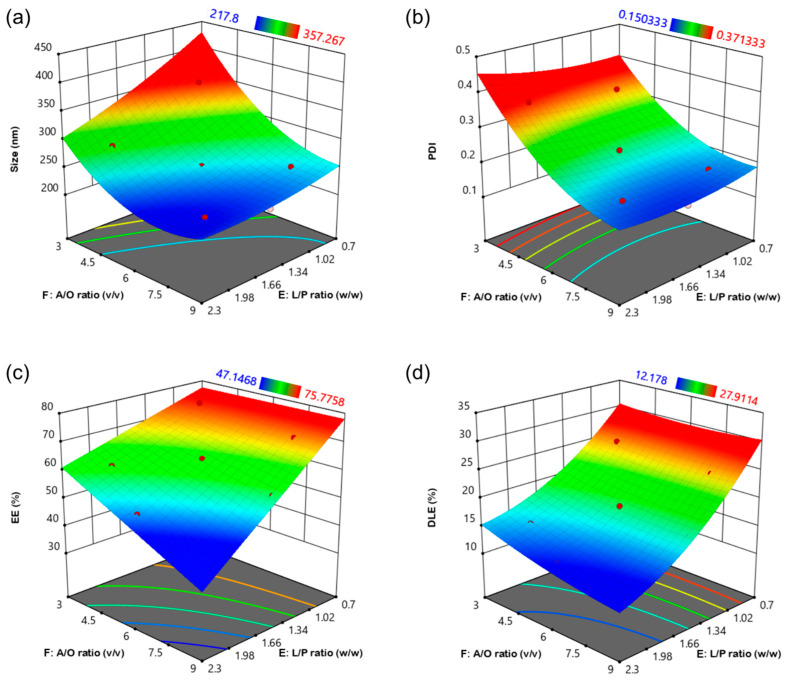
Response surface plots of particle size, PDI, EE and DLE responses of LPHNs. The figure shows the effects of A/O ratio vs. L/P ratio on particle size (**a**), PDI (**b**) EE (**c**), and DLE (**d**) responses of the prepared LPHNs.

Increasing the A/O volume ratio from 4/1 to 8/1 *v*/*v* resulted in a significant reduction in both particle size and PDI of the formed LPHNs, by approximately 100 nm and 0.15, respectively (Figure 5a,b). This trend can be explained by the mechanism of the lipid shell formation during the ethanol injection method. Ethanol injection is basically a solvent displacement/nanoprecipitation self-assembly process. When the organic phase (ethanol + lipids) is injected into a large volume of aqueous solution, rapid ethanol dilution occurs, leading to a sudden drop in the lipid solubility due to the increased solution polarity. As lipid solubility decreases, lipid molecules start to precipitate and self-assemble in the form of bilayer phospholipid fragments, with the hydrophilic head groups facing the aqueous medium and the tails directed towards each other to minimize the unfavorable interactions. These initial bilayer fragments retain exposed hydrophobic lateral ends, which drive their subsequent side-by-side fusion to minimize unfavorable interactions with the surrounding aqueous medium. Finally, the growing lipid bilayer fragments form a completely closed phospholipid bilayer vesicle [28]. The vesicle size has been reported to be dependent on the ethanol concentration in the aqueous phase after injection. Increasing the ethanol ratio promotes the formation of larger bilayer fragments and prolongs the vesicle growth time, producing larger vesicles. In contrast, lower ethanol content suppresses bilayer growth, facilitating rapid formation of smaller, more uniform vesicles [41].

On the other hand, increasing the L/P mass ratio from 1/1 to 2/1 *w*/*w* significantly reduced particle size of the LPHNs (Figure 5a). During the ethanol injection step, polymer concentration was fixed at 1.24 mg/mL as optimized earlier in this work. So, increasing the L/P mass ratio basically means increasing the lipid concentration. In contrast to our observations, multiple studies have reported an increase in particle size of liposomes formed by the ethanol injection method upon increasing phospholipid concentration above 40 mg/mL in ethanol [42,43,44]. For example, Stano et al. (2004) reported that increasing POPC (1-palmitoyl-2-oleoyl-sn-glycero-3-phosphocholine) lipid concentration in ethanol from 10 mM (~7.6 mg/mL) to 100 mM (~76 mg/mL) led to a significant increase in particle size of the formed liposomes from ~50 nm to more than 400 nm at an injection ratio of 1:20 *v*/*v* [43]. This effect is explained by Sebaaly et al. (2016) that higher phospholipid concentrations in organic phase led to formation of more bilayer fragments, increasing the possibility of fusion into larger vesicles [45]. However, an opposite trend has been reported in LPHNs by Heba et al. where they described formation of smaller LPHNs when higher lipid amounts were used, which supports our current findings [46]. This counterintuitive effect observed in LPHN formulations can be attributed to the low lipid concentrations used (<2.5 mg/mL of the final formulation) compared to high concentrations (>10 mg/mL) investigated in the liposomal formulations mentioned above. Moreover, increasing lipid concentration within our studied range (from 1.24 to 2.48 mg/mL) possibly improves shell compactness around the preformed polymer cores, resulting in a more compact and uniformly coated LPHNs.

It is worth mentioning that visible particle aggregation was observed in the formulation prepared at A/O ratio of 3.17 *v*/*v* and L/P ratio of 1.5 *w*/*w* (Table 5), which was considered as a formulation failure. Statistical analysis indicated that only A/O ratio had a significant effect on the likelihood of formulation failure (*p* = 0.0253), with higher failure probabilities being linked to lower A/O ratio, specifically below 4/1 *v*/*v*. Mechanistically, a reduced A/O ratio increases the relative ethanol content during injection, potentially forming fewer but larger lipid bilayer fragments. These larger fragments may enclose multiple polymeric cores upon their fusion in a single lipid shell, leading to the formation of oversized, unstable particles prone to aggregation and eventual sedimentation.

For the ZP response, all the LPHN formulations exhibited more negative ZP values compared to the uncoated polymeric cores (~−40 vs. ~−20 mV, respectively) (Table 5 and Table 3, respectively), confirming successful lipid shell association. In addition, all the formulations had ZPs below −30 mV, indicating good colloidal stability. However, the data could not be fitted to any statistically meaningful model, as indicated by poor fit statistics and nonsignificant model terms. This limitation is possibly due to the narrow measurement range and minimal variability of the measured values. Therefore, ZP response was excluded from subsequent analysis.

#### 3.2.2. Effect of the Studied Factors on LYZ EE and DLE in LPHNs

LYZ encapsulation data were fitted to a two-factor interaction (2FI) model with R^2^ of 0.9575, adjusted R^2^ of 0.9321, and predicted R^2^ of 0.8215, indicating good model fitting. The EE across the LPHN formulations ranged from 47.15% to 75.78%. ANOVA showed a significant model with both A/O ratio and L/P ratio having significant effects on LYZ EE in LPHNs. These effects are summarized in equation (Equation (9)). Increasing the A/O ratio or L/P ratio significantly reduced LYZ encapsulation (Figure 5c), likely due to the increased lipid shell compactness and the reduced particle size (Figure 5a). During the lipid shell formation, fusion of lipid bilayer fragments and their subsequent adherence to the preformed Eud-LYZ cores might result in partial displacement of the superficially bound LYZ molecules from Eud-LYZ NPs. This displacement becomes more pronounced at higher A/O and L/P ratios, where tighter and more compact shell formation promotes greater removal of loosely associated LYZ, leading to substantial reduction in LYZ encapsulation in the formed LPHNs.

(9) EE=63.35−9.02E−3.97F−2.59EF

where (*E*) is L/P ratio, and (*F*) is A/O ratio.

Drug loading content is considered one of the most important factors in development of protein-loaded nanocarriers. Achieving high protein loading content is required for delivery of therapeutically effective doses with minimal nanocarrier amount, which improves the overall nanocarrier potency and clinical applicability [47]. LYZ loading efficiencies were calculated for each of the design formulations and the obtained data were fitted to a quadratic model with good model fitting indicated by the high R^2^ of 0.9955, adjusted R^2^ of 0.9880, and predicted R^2^ of 0.9614. Statistical analysis revealed that both L/P mass ratio and A/O volume ratio had significant effects on LYZ DLE in LPHNs however L/P mass ratio was more significant and showed more profound effects as indicated from the response equation (Equation (10)).

(10) $$DLE=17.93-5.58E-0.918F-0.5316EF+1.15E^{2}+0.3139F^{2}$$

where (*E*) is L/P ratio, and (*F*) is A/O ratio.

The calculated DLE values ranged from 12.18% to 27.91%, with the highest values observed at the lowest L/P mass ratio (Figure 5d). Because DLE represents the percentage of LYZ relative to the total nanoparticle mass, lowering the L/P ratio reduces the overall particle mass and consequently increases the proportional LYZ content, resulting in higher DLE. The high DLE achieved in this work (up to 28% *w*/*w*) by far exceeds the values reported for typical LPHN systems (generally between 5 and 15% *w*/*w*) [12]. This highlights the potential of the developed LPHN system to deliver higher protein doses within a clinically feasible formulation volume, which is particularly advantageous for buccal delivery where dosage form size is inherently limited.

#### 3.2.3. Selection and Validation of an Optimized LPHN Formulation

**Figure 6:**
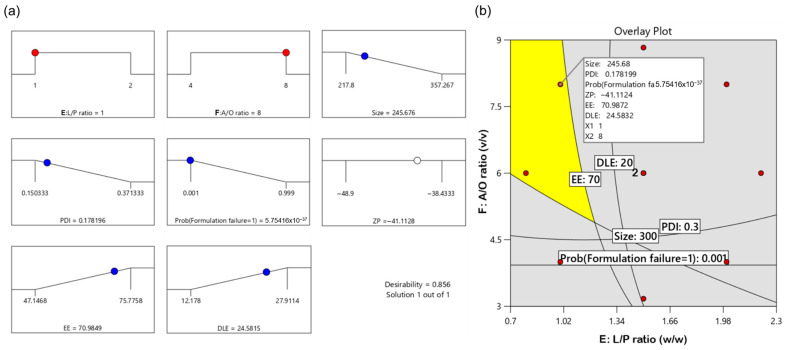
Numerical and graphical optimization of LHPNs. The figure shows the numerical optimization ramps representing the optimal factor conditions and the predicted responses (**a**), and the design space region (in yellow color) within the overall model space (**b**).

To select an optimized LPHN formulation, the numerical optimization function was used, and the selection criteria were set to minimize particle size, PDI, and formulation failure probability, while maximizing LYZ EE and DLE. The software suggested one solution at A/O ratio of 8/1 *v*/*v*, and L/P ratio of 1/1 *w*/*w*, where the particle size, PDI, Formulation failure, ZP, EE, and DLE were predicted to be 245.7 nm, 0.178, 0.000, −41.11 mV, 70.98%, and 24.58%, respectively (Figure 6a). These predicted values were experimentally validated by preparing three independent batches of the optimized LPHN formulation and comparing the experimentally obtained results with the predicted responses. Experimental validation of the predicted optimized formulation showed that the results obtained were in good agreement with the predicted values, with all the measured responses falling within the 95% confidence intervals, confirming a successful LPHN optimization process (Table 6).

In addition, the graphical optimization function was applied for design space determination. The upper limits for particle size, PDI, Formulation failure, and ZP were set as 300 nm, 0.3, 0.001, and −30 mV, while the lower limits of EE and DLE were set as 70% and 20%, respectively. The overlay plot showing the design space comprising all the set limits is demonstrated in Figure 6b.

Moreover, incorporation of NaDC in the optimized LPHN formulation in a ratio of 0.2 *w*/*w* of the PC content produced an additional variant with very close physicochemical properties to the original formulation. The new NaDC-LPHN formulation had comparable properties to the optimized LPHN with a slightly smaller particle size (Table 6). This modification was introduced to improve the formulation permeability across the buccal lining through the well-established permeation enhancement capabilities of the NaDC component [48,49].

### 3.3. Effect of Ionic Strength on LYZ Release

**Figure 7:**
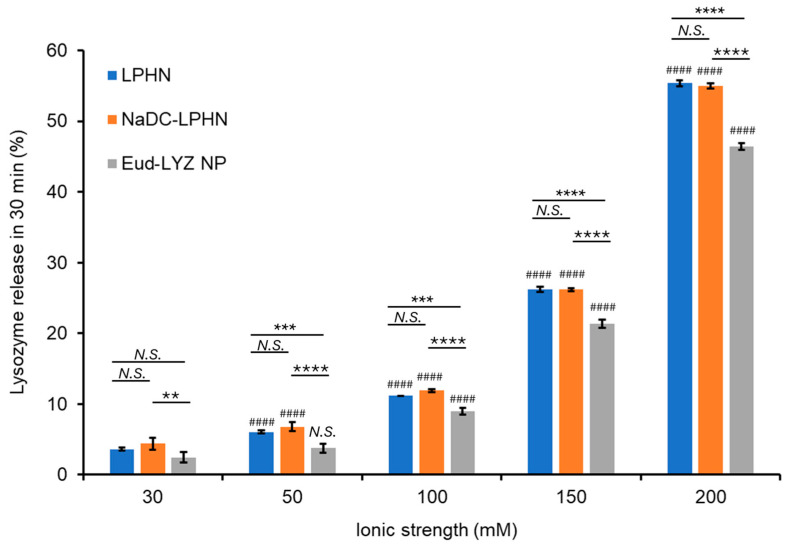
The effect of ionic strength on LYZ release from LPHNs (blue), NaDC-LPHNs (orange), and Eud-LYZ NPs (gray) after incubation for 30 min in release media with different ionic strengths. Values are represented as Mean ± SD, (*n* = 3). #### *p* < 0.0001 vs. 30 mM ionic strength. ** *p* < 0.01, *** *p* < 0.001, **** *p* < 0.0001, and *N.S. p* > 0.05.

LYZ release from Eud–LYZ NPs, optimized LPHNs, and NaDC-LPHNs showed a clear dependence on the ionic strength of the release medium. Minimal release (less than 10%) was observed at lower ionic strengths (30 and 50 mM), whereas increasing the ionic strength to 200 mM significantly increased LYZ release, reaching approximately 55% within 30 min of incubation (Figure 7). These findings are consistent with a previous study by our group where a salt concentration-dependent dissociation of LYZ from SDS-LYZ complex was observed [29]. This behavior is characteristic of systems that are stabilized mainly by electrostatic interactions. In the present formulations, LYZ is loaded into the polymer core via strong attractions between the highly cationic LYZ and the anionic polymer Eud-L100, a mechanism already evident from the pH-dependent encapsulation trends described earlier. At low ionic strengths, fewer free ions are attracted to the charged functional groups of the protein and the polymer, leaving the electrostatic attractions un-affected and preserving the integrity of the protein–polymer complex with minimal LYZ dissociation.

However, as ionic strength increases, the abundance of free mobile ions in the media (mainly Na^+^ and Cl^−^) induces electrostatic charge screening where both the range and magnitude of the electrostatic interactions are reduced [50]. The abundant mobile ions attract to the charged groups on LYZ and Eud-L100, shielding the effective surface charges and weakening the electrostatic attractions that stabilize the protein–polymer complex. This effect strongly weakens the association between LYZ and Eud-L100, lowering the dissociation energy and allowing LYZ to diffuse out of the nanoparticles.

Although it was expected that the presence of the lipid shell in LPHNs would provide an additional diffusional barrier and thereby delay LYZ release, the opposite was true, with both LPHN and NaDC-LPHN formulations showing higher release percentages than the Eud–LYZ NPs across all ionic strengths. The exact mechanism is not fully understood, but it can be concluded that LYZ release is predominantly driven by complex dissociation and nanoparticle destabilization rather than by simple diffusion. Furthermore, the lipid shell might somehow intensify rather than mitigate the ionic strength-induced charge-screening effect, thereby facilitating LYZ release from LPHN and NaDC-LPHN, resulting in higher release percentages compared to the Eud-LYZ NPs.

### 3.4. In Vitro LYZ Release

**Figure 8:**
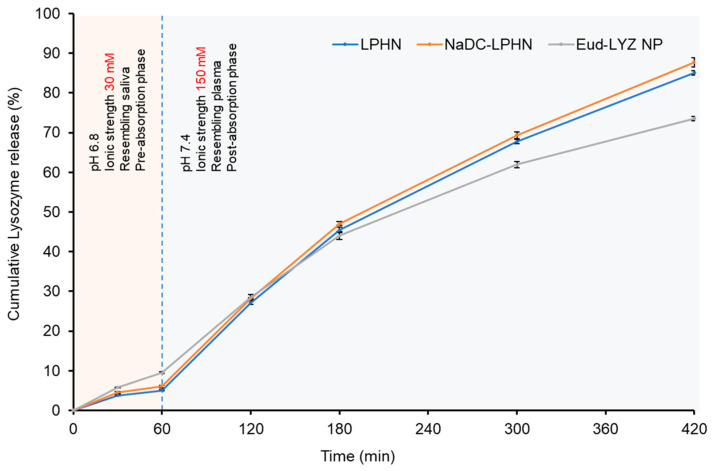
Cumulative LYZ release from LPHNs (blue line), NaDC-LPHNs (orange line), and Eud-LYZ NPs (gray line) in two sequential release media conditions: saliva-like medium (0–60 min), and plasma-like medium (60–420 min). Values are represented as Mean ± SD, (*n* = 3).

Cumulative LYZ release from LPHNs, NaDC-LPHNs, and Eud-LYZ NPs was evaluated in two sequential buffers simulating pre-absorption and post-absorption conditions. During the first 60 min, all formulations showed minimal LYZ release, with cumulative release values remaining below 10% (5.04 ± 0.17% for LPHN, 6.02 ± 0.35% for NaDC-LPHN, and 9.42 ± 0.24% for Eud-LYZ NPs) at pH 6.8 and an ionic strength of 30 mM (Figure 8). This low release level reflects the strong electrostatic attractions between LYZ and the Eud-L100 polymer at low ionic strength conditions, where the electrostatically stabilized complex is minimally affected by the low concentration of mobile ions.

After changing the release medium to pH 7.4 and an ionic strength of 150 mM, a sharp increase in LYZ release was observed in all formulations. During the first hour after the medium change (60–120 min), cumulative release rapidly increased to ~28% (precisely 27.14 ± 0.41% for LPHN, 28.20 ± 0.15% for NaDC-LPHN, and 28.48 ± 0.77% for Eud-LYZ NPs), showing ionic strength-responsive behavior. This rapid increase in LYZ release is consistent with the charge screening effect discussed earlier, where a higher concentration of the mobile ions (mainly Na^+^ and Cl^−^) can effectively shield the surface charges of LYZ and Eud-L100. Once surface charges are screened by the abundant mobile ions, electrostatic interactions that stabilize the protein–polymer complex are greatly weakened and complex dissociation becomes easier, facilitating faster LYZ diffusion out of the nanoparticles.

The three formulations maintained a greater release rate in the second medium (plasma-mimicking) compared to the first (saliva-mimicking) and the differences between formulations became clearer as the release study progressed. By the end of the test after 420 min, NaDC-LPHNs showed the highest cumulative release (87.67 ± 1.12%) followed by LPHN (85.02 ± 0.52%), while Eud-LYZ NPs displayed a comparatively slower release profile (73.53 ± 0.50%). This variation can be attributed to the structural differences between the formulations. NaDC-LPHN and LPHN formulations have lipid shells formed of PC with or without NaDC, which unexpectedly, may promote system destabilization at high ionic strength conditions, causing higher cumulative LYZ release compared to the uncoated polymeric Eud-LYZ NPs. The slightly higher release from NaDC-LPHNs may arise from the increased membrane fluidity caused by incorporation of NaDC [51].

Overall, these results demonstrate that the developed L PHN formulations can maintain minimal LYZ leakage under buccal-like ionic conditions while enabling rapid and effective release upon transition to plasma-like ionic conditions. This ionic-strength responsiveness is a key advantage for buccal delivery, ensuring stability at the application site and efficient release following systemic absorption.

### 3.5. Storage Stability Test

**Figure 9:**
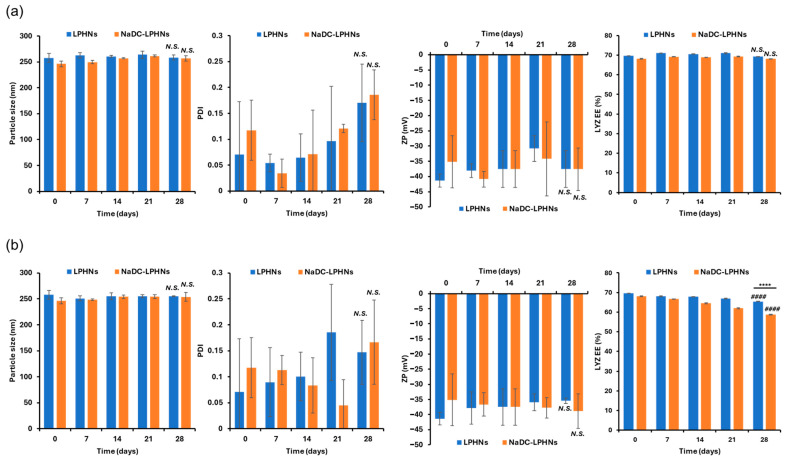
Storage stability of LPHNs and NaDC-LPHNs in different storage conditions. The figure shows particle size, PDI, ZP, and EE of LPHNs and NaDC-LPHNs over time at 4 °C (**a**) and 25 °C (**b**). Data are represented as Mean ± SD, (*n* = 3). **** *p* < 0.0001. #### *p* < 0.0001, and *N.S. p* > 0.05 vs. time zero.

The storage stability of LPHN and NaDC-LPHN formulations was observed over 28 days of storage at 4 and 25 °C. A colloidally stable nanoparticle suspension should exhibit constant particle size, PDI, ZP, and EE throughout the study period with minimal changes. Results presented in Figure 9 show that both formulations, LPHN and NaDC-LPHN, exhibited constant particle size, ZP, and EE at 4 °C with a slight increase in PDI values (*p* > 0.05) over the study duration, indicating excellent colloidal stability. In contrast, while particle size, PDI, and ZP showed insignificant changes at 25 °C, a significant reduction in LYZ EE was observed in both formulations after 28 days. However, the reduction in EE was higher in NaDC-LPHN compared to LPHN, likely due to the presence of NaDC which increases the lipid shell fluidity and results in more LYZ leakage [51]. LPHN had a starting EE of 69.64 ± 0.11% which decreased to 69.25 ± 0.13% (*p* > 0.05), and 65.42 ± 0.13% (*p* < 0.0001) at 4 and 25 °C, respectively, after 28 days. On the other hand, NaDC-LPHN had an initial EE of 68.14 ± 0.16% which decreased over time to 68.13 ± 0.08% at 4 °C (*p* > 0.05), and to 58.70 ± 0.21% (*p* < 0.0001) at 25 °C. The stability of these formulations at lower temperatures could be attributed to the immobilization of lipids within the lipid shell, preventing LYZ leakage or other structural changes in LPHNs. In addition, the presence of the rigid polymer, Eud-L100, in the internal core is believed to have a major role in preserving the structural integrity of LPHN formulations as indicated by the minimal changes in particle size, PDI, and ZP after storage at 4 and 25 °C, especially when compared to other lipid and surfactant-based formulations [52,53,54].

Overall, the developed formulations showed excellent colloidal stability when stored at 4 °C with minimal changes in their physical attributes and protein EE. Although stability was lower at room temperature compared to 4 °C, both formulations exhibited good structural stability with no substantial changes in particle size, PDI, or ZP.

### 3.6. FT-IR Spectroscopy

**Figure 10:**
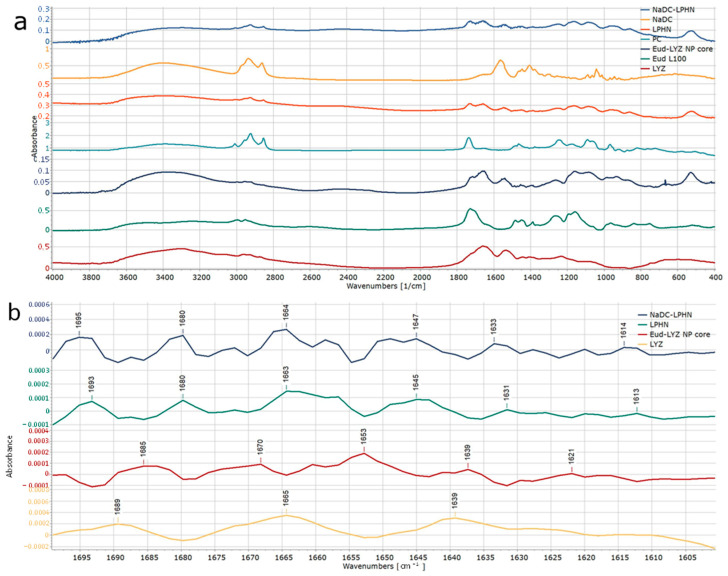
(**a**) FT-IR spectra of NP cores, LPHNs, NaDC-LPHNs, free LYZ, Eud-L100, PC lipid, and NaDC; and (**b**) the inverted second derivative of the amide I region of the spectra of NP cores, LPHNs, NaDC-LPHNs, free LYZ.

Figure 9 displays the FT-IR spectra of raw materials, the NP core as well as LPHNs and NaDC-LPHNs to confirm LYS encapsulation and investigate the possible changes in LYZ secondary structure. Eud-L100 shows a characteristic band at 1729 cm^−1^ corresponding to esterified C=O stretching, and two peaks at 1265 cm^−1^ and 1158 cm^−1^ representing C–O vibrations due to ester linkage. In the case of NP cores, the C=O stretching is shifted to 1715 cm^−1^ which may be an indication of successful complexation with LYZ. Nevertheless, in case of LPHNs and NaDC-LPHNs, a reorganization may be observed, which may support the observation of LYZ leakage due to the weakening electrostatic interactions. In case of LPHN and NaDC-LPHNs minor shifts in ester band to 1250 cm^−1^ and 1249 cm^−1^, respectively, may indicate the involvement of this molecular part to the core–shell interactions. The most important peaks in LYZ spectrum belong to the amide I (1700–1600 cm^−1^) and amide II (1600–1500 cm^−1^) bands [55,56]. As shown in Figure 10a, the characteristic amide I band, corresponding to C=O stretching vibration and amide II band, corresponding to N–H bending vibrations of LYZ were observed at 1660 cm^−1^ and 1540 cm^−1^, respectively. The slight left shift in amide II band to 1542 cm^−1^, 1548 cm^−1^ and 1550 cm^−1^ in the case of NP cores, LPHNs and NaDC-LPHNs, respectively also indicating the presence of Eud-LYZ interactions. Nevertheless, therapeutic proteins must retain their native structure to be functional, and changes in their secondary structure can considerably alter their therapeutic activity. To better understand the effect of interactions on the structure of LYZ, the protocol of Yang et al. was followed [54]. Figure 10b displays the inverted second derivative of the amide I region. It is clearly visible that LYZ has three characteristic peaks in the region at 1639 cm^−1^, 1665 cm^−1^ and 1689 cm^−1^ representing β-sheet, α-helix and β-turn structural elements, respectively. In the case of the NP core, the right shift in the α-helix signal to 1653 cm^−1^ indicates the transformation of the helical structure to random form due to the strong polymer-protein interactions. Nevertheless, a considerable reorganization may be observed in the spectra of LPHN and NaDC-LPHN where the α-helix signal appears in its original position while β-sheet and β-turn signals are shifted to 1645 cm^−1^ and 1680 cm^−1^, respectively. This may indicate the involvement of apolar molecular parts to protein-lipid and polymer-lipid interactions, and confirms the dynamic nature of the molecular structure of the developed LPHN and NaDC-LPHN formulations based on secondary forces, and indicate that LYZ could regain its secondary structure after the disassembly of NPs [57].

These results confirm that the developed LPHN formulations can preserve the structural integrity of encapsulated LYZ and suggest that our applied preparation methods are protein-friendly and can be utilized for other labile drugs.

## 4. Conclusions and Future Directions

In this study, we developed and optimized an ionic strength-responsive LPHN system designed to enhance the buccal delivery of LYZ. Through integrated ionic gelation and ethanol injection methods, optimal Eud-LYZ cores and LPHNs were formulated with desirable physicochemical properties and high encapsulation and protein loading efficiencies. The optimized LPHN formulation demonstrated a uniform particle size suitable for buccal delivery, while maintaining high-protein EE and DLE, which is critical for achieving higher doses with the smallest possible volume. The optimized formulations exhibited minimal LYZ leakage under salivary-like ionic conditions while enabling rapid and substantial release upon exposure to plasma-like ionic strengths, allowing for better protection of the loaded protein in the pre-absorption buccal environment. The achieved high EE and DLE, and incorporation of a permeation enhancer, together with the minimal pre-absorption leakage are expected to significantly contribute to the improved overall bioavailability and therapeutic efficacy of the encapsulated protein therapeutics. Collectively, these findings demonstrate that the developed LPHNs offer an effective novel strategy for site-specific protection and systemic-triggered release of protein drugs, highlighting their strong potential as a next-generation buccal delivery platform.

Despite the strengths of this work, there are some limitations that should be acknowledged. First, the current work discussed the development of ionic strength-responsive LPHNs for buccal protein delivery primarily from a technological perspective, without providing mechanistic information on how these formulations interact with biological membranes in the buccal mucosa. In addition, the lack of biological evaluation of the encapsulated protein’s stability and activity after formulation, as well as the absence of permeability and transport studies, limits the assessment of the therapeutic potential of the developed systems.

However, future in vitro, ex vivo, or in vivo buccal permeation studies as well as storage stability and enzymatic activity studies are still needed to evaluate permeation enhancement, colloidal stability, and protection efficiency of the designed LPHN system, especially in comparison with other conventional buccal delivery platforms. Nevertheless, due to the complexity of the field, the results of these proposed investigations will be published in a separate manuscript. Moreover, incorporating these formulations into mucoadhesive buccal films or sublingual tablets could further improve their buccal residence time and absorption efficiency, unlocking the true potential for their clinical translation.

## Figures and Tables

**Table 1:** The selected factors and responses of the two-level factorial design used for polymer core optimization.

Factors	Lower Limit	Upper Limit	Rationale
A: Eud-L100 (mg/mL)	0.5	1.5	To avoid aggregation at higher concentrations and formulation failure below the lower limit.
B: Eud/LYZ ratio (*w*/*w*)	1	2	≥1 *w*/*w* to avoid incomplete complexation and reduced EE due to insufficient Eud-L100 ratio. ≤2 *w*/*w* to avoid unnecessary reduction in DLE due to higher Eud-L100 ratio.
C: pH	6.8	8.8	Eud-L100 solubility is greatly reduced under pH 6.8, while at pHs above 8.8, LYZ becomes less ionized and complexation efficiency is reduced [30,31].
D: Temperature (°C)	25	50	This range allows investigating the temperature effect without harming LYZ integrity [32].
**Responses**			
Size (nm)			
PDI			
ZP (mV)			
EE (%)			

**Table 2:** The selected factors and responses of the central composite design used for lipid shell optimization.

Factors	Lower Limit	Upper Limit	Rationale
E: L/P ratio (*w*/*w*)	1	2	Lower lipid ratio may cause incomplete particle coating, while higher ratio may lead to formation of separate lipid vesicles or multilamellar lipid coats.
F: A/O ratio (*v*/*v*)	4	8	This range covers most of the reported values of A/O volume ratios in ethanol injection method. Lower ratios are associated with larger particle sizes [33].
**Responses**			
Size (nm)			
PDI			
Formulation failure			
ZP (mV)			
EE (%)			
DLE (%)			

**Table 3:** Different polymeric Eud-LYZ NP formulations with their measured responses based on factorial design.

Run	Eud-L100 (mg/mL)	Eud/LYZ (*w*/*w*)	pH	Temperature (°C)	Size (nm)	PDI	ZP (mV)	EE (%)
1	1.5	1	8.8	25	433.8 ± 9.0	0.199 ± 0.244	−36.53 ± 7.15	40.90 ± 4.13
2	0.5	1	8.8	50	255.9 ± 107.5	0.397 ± 0.078	−4.37 ± 3.04	−0.57 ± 0.77
3	1.5	1	6.8	50	249.9 ± 5.1	0.079 ± 0.028	−29.00 ± 1.97	67.28 ± 2.49
4	1	1.5	7.8	37.5	318.9 ± 57.5	0.322 ± 0.180	−15.60 ± 3.44	8.96 ± 1.76
5	0.5	2	6.8	50	217.3 ± 3.9	0.061 ± 0.027	−0.53 ± 0.70	22.18 ± 0.69
6	1.5	2	8.8	50	363.1 ± 8.2	0.172 ± 0.115	−26.67 ± 2.39	2.26 ± 0.64
7	0.5	2	8.8	25	526.0 ± 34.2	1.299 ± 0.399	−14.83 ± 3.55	−0.24 ± 0.43
8	0.5	1	6.8	25	266.3 ± 13.4	0.246 ± 0.032	−2.37 ± 1.95	59.66 ± 0.49
9	1.5	2	6.8	25	228.2 ± 6.9	0.261 ± 0.043	−9.57 ± 4.66	47.08 ± 0.37

**Table 4:** Predicted versus observed responses of the optimized Eud-LYZ NP formulation.

Responses	Predicted	95% PI Low	95% PI High	Observed 1	Observed 2	Observed 3	Mean ± SD
Size (nm)	235.1	222.1	249.2	239.8	245.6	238.8	242.2 ± 4.8
PDI	0.061	−0.127	0.249	0.214	0.105	0.104	0.141 ± 0.063
ZP (mV)	−19.74	−20.99	−18.50	−22.67	−18.87	−18.73	−20.09 ± 2.24
EE (%)	61.55	34.78	88.32	68.79	70.23	69.06	69.36 ± 0.77

**Table 5:** Different LPHN formulations with their measured responses based on central composite design.

Run	L/P Ratio (*w*/*w*)	A/O Ratio (*v*/*v*)	Size (nm)	PDI	F.F.	ZP (mV)	EE (%)	DLE (%)
1	1	8	253.4 ± 0.9	0.184 ± 0.082	0	−40.13 ± 7.81	71.87 ± 0.25	24.62 ± 0.07
2	2	4	287.6 ± 6.1	0.371 ± 0.069	0	−48.90 ± 1.90	61.42 ± 0.24	15.30 ± 0.05
3	2	8	224.1 ± 8.3	0.190 ± 0.014	0	−47.53 ± 3.30	47.15 ± 0.04	12.18 ± 0.01
4	1.5	6	252.9 ± 4.3	0.237 ± 0.070	0	−45.43 ± 0.15	58.11 ± 0.10	17.19 ± 0.03
5	1	4	357.3 ± 11.3	0.338 ± 0.017	0	−45.20 ± 1.79	75.78 ± 0.04	25.62 ± 0.01
6	1.5	3.17	6395.6 ± 2269.8 *	2.279 ± 1.339 *	1	−41.07 ± 0.25	68.52 ± 0.02	19.66 ± 0.01
7	1.5	8.83	221.5 ± 2.9	0.150 ± 0.014	0	−42.87 ± 3.68	58.89 ± 0.10	17.38 ± 0.03
8	1.5	6	250.3 ± 10.3	0.213 ± 0.166	0	−38.43 ± 4.44	64.29 ± 0.15	18.67 ± 0.03
9	0.79	6	286.0 ± 10.0	0.228 ± 0.052	0	−41.13 ± 0.72	75.42 ± 0.02	27.91 ± 0.01
10	2.21	6	217.8 ± 2.9	0.226 ± 0.009	0	−43.70 ± 4.17	52.03 ± 0.09	12.47 ± 0.02

* These values were excluded from data analysis due to severe aggregation; F.F. is formulation failure response.

**Table 6:** Predicted versus observed responses of the optimized LPHNs and physicochemical properties of NaDC-LPHN formulation.

Responses	Predicted	95% PI Low	95% PI High	Observed 1	Observed 2	Observed 3	Mean ± SD	NaDC-LPHN
Size (nm)	245.7	224.3	267.1	258.4	255.5	257.7	257.2± 1.5	246.0 ± 5.7
PDI	0.178	0.133	0.224	0.261	0.213	0.071	0.182 ± 0.099	0.117 ± 0.058
F.F.	0.000	0.000	0.000	0.000	0.000	0.000	0.000 ± 0.000	0.000
ZP (mV)	−41.11	−45.78	−36.44	−42.33	−34.93	−41.37	−39.54 ± 4.02	−35.17 ± 8.57
EE (%)	70.98	64.35	77.62	70.03	70.00	69.64	69.89 ± 0.22	68.14 ± 0.16
DLE (%)	24.58	22.63	26.54	24.14	24.14	24.04	24.11 ± 0.06	23.65 ± 0.04

## Data Availability

Data is available upon request from the corresponding author. The datasets presented in this article are not readily available because the data are part of an ongoing study that requires further time until the raw data can be transformed to publicly accessible file formats, and the file names and nomenclature used in the various measurements would be unified and the correct metadata could be provided to enable the correct submission of the raw files into a publicly available data repository. Until that time, requests to access the datasets should be directed to the corresponding author (sovany.tamas@szte.hu).

## Supplementary Materials

The following supporting information can be downloaded at: https://www.mdpi.com/article/10.3390/pharmaceutics18060719/s1, Figure S1: Visual appearance of different LYZ concentrations in different pH conditions; Figure S2: Scatter plot of predicted versus observed data for particle size response of Eud-LYZ NPs; Figure S3: Scatter plot of predicted versus observed data for PDI response of Eud-LYZ NPs; Figure S4: Scatter plot of predicted versus observed data for ZP response of Eud-LYZ NPs; Figure S5: Scatter plot of predicted versus observed data for EE response of Eud-LYZ NPs; Figure S6: Scatter plot of predicted versus observed data for particle size response of LPHNs; Figure S7: Scatter plot of predicted versus observed data for PDI response of LPHNs; Figure S8: Scatter plot of predicted versus observed data for formulation failure response of LPHNs; Figure S9: Scatter plot of predicted versus observed data for ZP response of LPHNs; Figure S10: Scatter plot of predicted versus observed data for EE response of LPHNs; Figure S11: Scatter plot of predicted versus observed data for DLE response of LPHNs; Table S1: Build information for polymer core optimization design; Table S2: Factors used for the 2-level factorial design; Table S3: ANOVA for selected factorial model of Eud-LYZ NPs’ particle size response; Table S4: ANOVA for selected factorial model of Eud-LYZ NPs’ PDI response; Table S5: ANOVA for selected factorial model of Eud-LYZ NPs’ ZP response; Table S6: ANOVA for selected factorial model of Eud-LYZ NPs’ EE response; Table S7: Constraints for optimization of Eud-LYZ NPs; Table S8: Eud-LYZ NPs optimization solutions suggested by the software based on the pre-defined criteria; Table S9: Coefficients Table summarizing the effects of different factors on the studied responses of Eud-LYZ NPs; Table S10: Build information for lipid shell optimization design; Table S11: Factors used for the central composite design; Table S12: ANOVA for Quadratic model of LPHNs’ particle size response; Table S13: ANOVA for Quadratic model of LPHNs’ PDI response; Table S14: Logistic Regression (Type III) for formulation failure response of LPHNs; Table S15: Fit Summary for ZP response of LPHNs; Table S16: ANOVA for Linear model of LPHNs’ ZP response; Table S17: Fit statistics for ZP response of LPHNs; Table S18: ANOVA for 2FI model of EE response of LPHNs; Table S19: ANOVA for Quadratic model of DLE response of LPHNs; Table S20: Constraints used for optimization of LPHNs; Table S21: LPHN optimization solutions suggested by the software based on pre-defined criteria; Table S22: Coefficients Table summarizing the effects of different factors on the studied responses of LPHNs.
