# Supplementary material for: Development and Optimization of Ionic Strength-Responsive Lipid–Polymer Hybrid Nanoparticles for Buccal Protein Delivery
Source: Pharmaceutics. 2026 Jun 11;18(6):719. doi: 10.3390/pharmaceutics18060719 (PMC13305947; doi:10.3390/pharmaceutics18060719)
Supplement: Supplementary file 1 [file pharmaceutics-18-00719-s001.zip › pharmaceutics-4291037-supplementary.pdf]

SUPPLEMENTARY MATERIALS

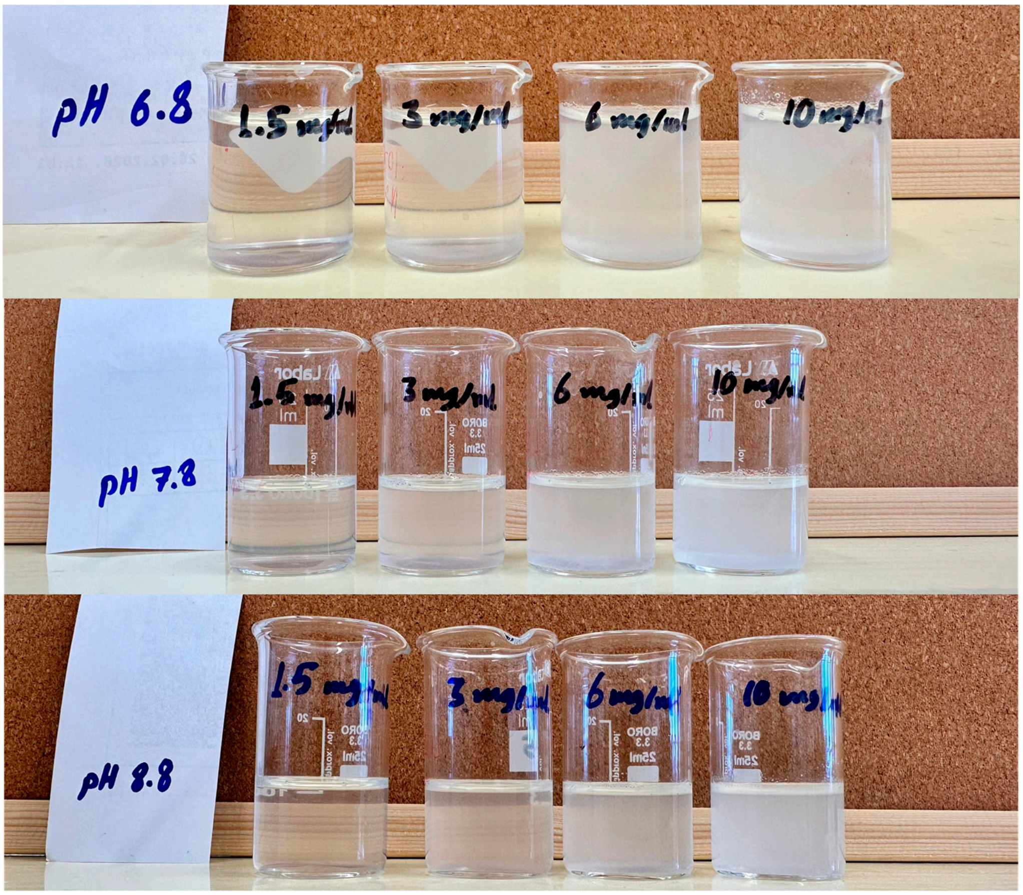

Figure S1. Visual appearance of different LYZ concentrations in different pH conditions.

Table S1. Build information for polymer core optimization design

**File Version**	13.0.5.0		
**Study Type**	Factorial	**Subtype**	Randomized
**Design Type**	2 Level Factorial	**Runs**	9.00
**Design Model**	Reduced 2FI	**Blocks**	No Blocks
**Center Points**	1.0000	**Build Time (ms)**	11.00

Table S2. Factors used for the 2-level factorial design

Factor	Name	Units	Type	SubType	Minimum	Maximum	Coded Low	Coded High	Mean	Std. Dev.
A	Eud. Conc	mg/mL	Numeric	Continuous	0.5000	1.50	-1 ↔ 0.50	+1 ↔ 1.50	1.0000	0.5000
B	Eud/LYZ ratio		Numeric	Continuous	1.0000	2.00	-1 ↔ 1.00	+1 ↔ 2.00	1.50	0.5000
C	pH		Numeric	Continuous	6.80	8.80	-1 ↔ 6.80	+1 ↔ 8.80	7.80	1.0000
D	Temperature	deg. C	Numeric	Continuous	25.00	50.00	-1 ↔ 25.00	+1 ↔ 50.00	37.50	12.50

Table S3. ANOVA for selected factorial model of Eud-LYZ NPs' particle size response

Source	Sum of Squares	df	Mean Square	F-value	p-value	
**Model**	88074.70	7	12582.10	8113.53	0.0085	significant
A-Eud. Conc	11.12	1	11.12	7.17	0.2275	
B-Eud/LYZ ratio	2072.61	1	2072.61	1336.51	0.0174	
C-pH	47601.55	1	47601.55	30695.70	0.0036	
D-Temperature	16937.20	1	16937.20	10921.90	0.0061	
AB	12285.28	1	12285.28	7922.12	0.0072	
AC	52.19	1	52.19	33.65	0.1087	
AD	9114.75	1	9114.75	5877.62	0.0083	
**Residual**	1.55	1	1.55			
**Cor Total**	88076.26	8				

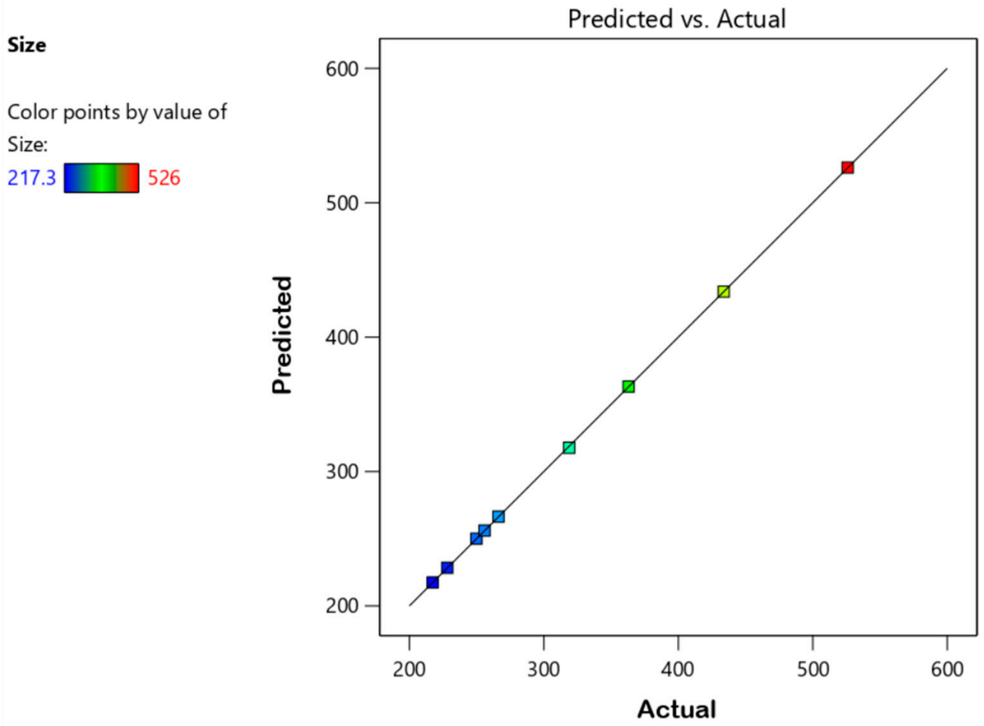

Figure S2. Scatter plot of predicted versus observed data for particle size response of Eud-LYZ NPs.

Table S4. ANOVA for selected factorial model of Eud-LYZ NPs' PDI response

Source	Sum of Squares	df	Mean Square	F-value	p-value	
**Model**	1.13	7	0.1617	585.70	0.0318	significant
A-Eud. conc	0.2088	1	0.2088	756.05	0.0231	
B-Eud/LYZ ratio	0.0950	1	0.0950	343.96	0.0343	
C-pH	0.2522	1	0.2522	913.24	0.0211	
D-Temperature	0.2103	1	0.2103	761.53	0.0231	
AB	0.0394	1	0.0394	142.81	0.0531	
AC	0.2302	1	0.2302	833.61	0.0220	
AD	0.0963	1	0.0963	348.71	0.0341	
**Residual**	0.0003	1	0.0003			
**Cor Total**	1.13	8				

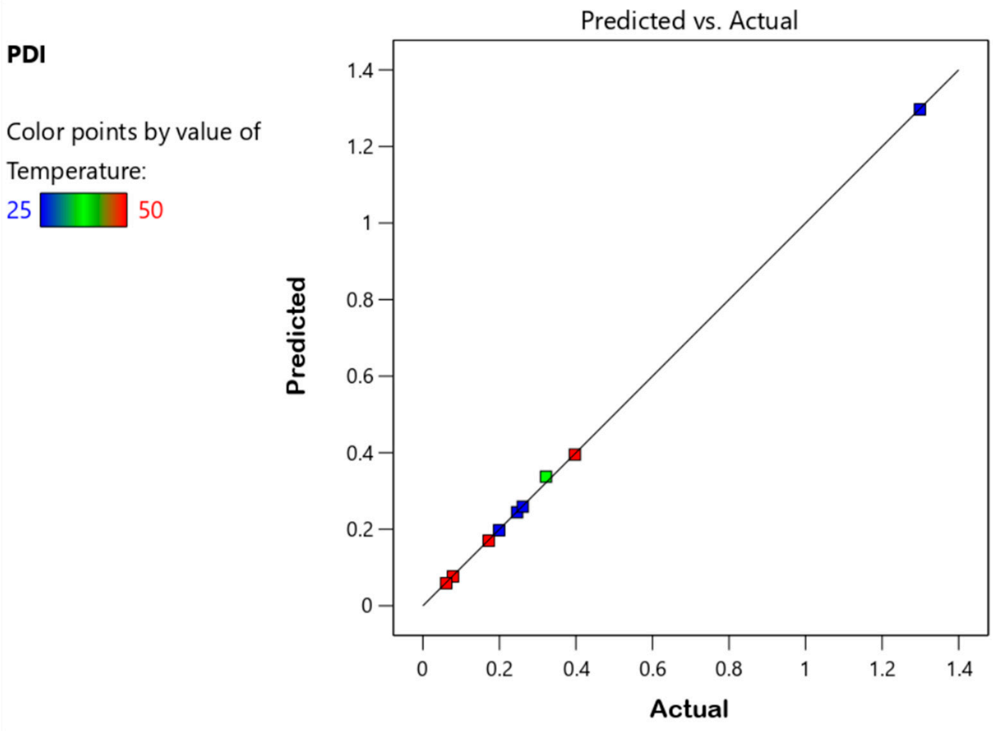

Figure S3. Scatter plot of predicted versus observed data for PDI response of Eud-LYZ NPs.

Table S5. ANOVA for selected factorial model of Eud-LYZ NPs' ZP response

Source	Sum of Squares	df	Mean Square	F-value	p-value	
**Model**	1305.43	7	186.49	15413.96	0.0062	significant
A-Eud. conc	793.35	1	793.35	65572.58	0.0025	
B-Eud/LYZ ratio	53.39	1	53.39	4412.76	0.0096	
C-pH	209.44	1	209.44	17311.04	0.0048	
D-Temperature	0.9339	1	0.9339	77.19	0.0722	
AB	179.87	1	179.87	14866.58	0.0052	
AC	8.68	1	8.68	717.47	0.0238	
AD	59.77	1	59.77	4940.08	0.0091	
**Residual**	0.0121	1	0.0121			
**Cor Total**	1305.44	8				

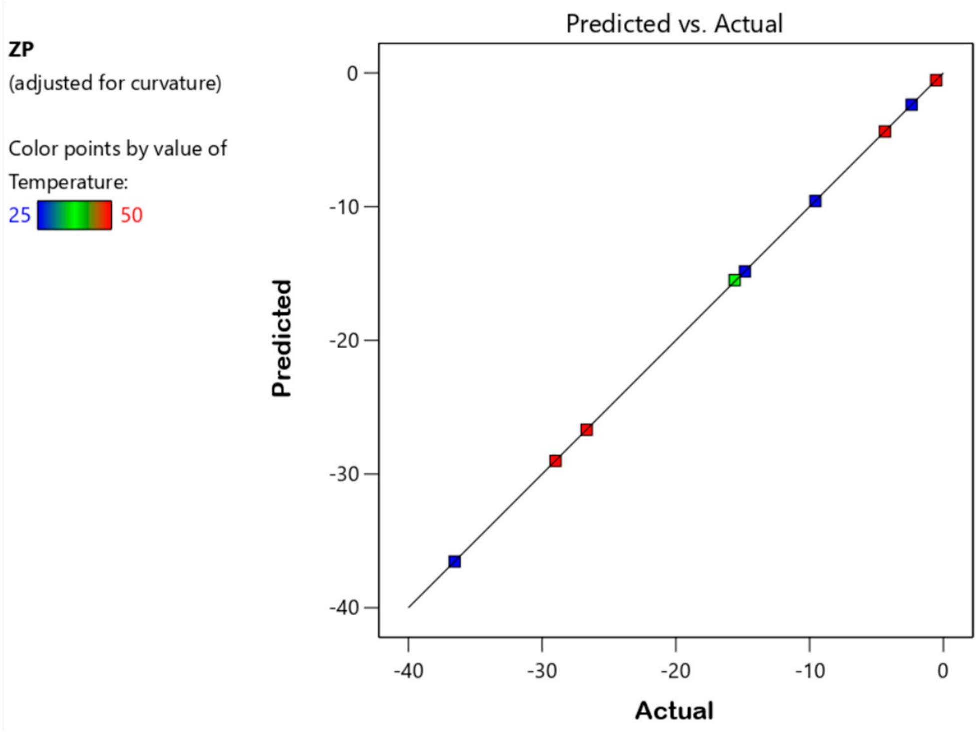

Figure S4. Scatter plot of predicted versus observed data for ZP response of Eud-LYZ NPs.

Table S6. ANOVA for selected factorial model of Eud-LYZ NPs' EE response

Source	Sum of Squares	df	Mean Square	F-value	p-value	
**Model**	5236.78	4	1309.20	10.29	0.0221	significant
A-Eud. conc	731.08	1	731.08	5.75	0.0746	
B-Eud/LYZ ratio	1151.53	1	1151.53	9.05	0.0396	
C-pH	2958.67	1	2958.67	23.26	0.0085	
D-Temperature	395.50	1	395.50	3.11	0.1526	
**Residual**	508.86	4	127.21			
**Cor Total**	5745.64	8				

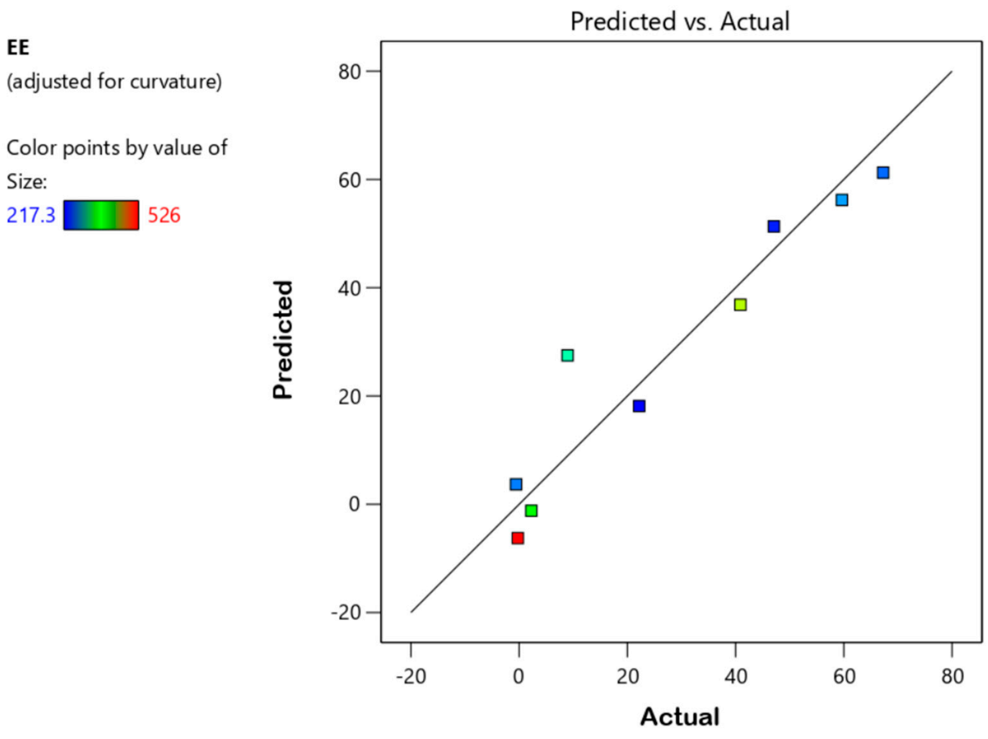

Figure S5. Scatter plot of predicted versus observed data for EE response of Eud-LYZ NPs.

Table S7. Constraints for optimization of Eud-LYZ NPs

Name	Goal	Lower Limit	Upper Limit	Lower Weight	Upper Weight	Importance
A:Eud. conc	is in range	0.5	1.5	1	1	3
B:Eud/LYZ ratio	is in range	1	2	1	1	3
C:pH	is in range	6.8	8.8	1	1	3
D:Temperature	is in range	25	50	1	1	3
Size	minimize	217.3	526	1	1	3
PDI	minimize	0.061	1.299	1	1	3
ZP	none	-36.5333	-0.533333	1	1	2
EE	maximize	-0.565773	67.2757	1	1	4

Table S8. Eud-LYZ NPs optimization solutions suggested by the software based on the pre-defined criteria

Number	Eud. conc	Eud/LYZ ratio	pH	Temperature	Size	PDI	ZP	EE	Desirability	
**1**	**1.239**	**1.000**	**6.800**	**40.618**	**235.119**	**0.061**	**-19.743**	**61.552**	**0.948**	**Selected**
2	1.173	1.000	6.800	40.256	229.944	0.050	-17.813	60.482	0.947	
3	1.362	1.000	6.802	38.688	249.917	0.100	-23.009	64.937	0.945	
4	1.423	1.029	6.800	35.461	258.316	0.131	-23.818	67.275	0.941	
5	1.484	1.001	6.800	37.704	260.798	0.126	-26.207	67.842	0.940	
6	1.500	1.119	6.800	34.207	259.985	0.152	-24.241	67.276	0.935	
7	1.322	1.093	6.800	44.620	233.308	0.061	-21.529	58.665	0.932	
8	1.236	1.000	6.864	49.999	217.309	-0.016	-20.731	54.987	0.923	
9	1.374	1.085	6.800	26.238	269.206	0.191	-20.598	70.173	0.915	

Table S9. Coefficients Table summarizing the effects of different factors on the studied responses of Eud-LYZ NPs

	Intercept	A	B	C	D	AB	AC	AD
**Size**	317.693	1.17917	**16.0958**	**77.1375**	**-46.0125**	**-39.1875**	2.55417	**33.7542**
**p-values**		0.2275	**0.0174**	**0.0036**	**0.0061**	**0.0072**	0.1087	**0.0083**
**PDI**	0.337333	**-0.161542**	**0.108958**	**0.177542**	**-0.162125**	-0.0702083	**-0.169625**	**0.109708**
**p-values**		**0.0231**	**0.0343**	**0.0211**	**0.0231**	0.0531	**0.0220**	**0.0341**
**ZP**	-15.4963	**-9.95833**	**2.58333**	**-5.11667**	0.341667	**4.74167**	**-1.04167**	**-2.73333**
**p-values**		**0.0025**	**0.0096**	**0.0048**	0.0722	**0.0052**	**0.0238**	**0.0091**
**EE**	27.5023	9.55953	**-11.9975**	**-19.2311**	-7.03122			
**p-values**		0.0746	**0.0396**	**0.0085**	0.1526			

p-value shading: **p < 0.05** 0.05 ≤ p < 0.1 p ≥ 0.1

Table S10. Build information for lipid shell optimization design

**File Version**	13.0.5.0		
**Study Type**	Response Surface	**Subtype**	Randomized
**Design Type**	Central Composite	**Runs**	10.00
**Design Model**	Quadratic	**Blocks**	2.00

Table S11. Factors used for the central composite design

Factor	Name	Units	Type	SubType	Minimum	Maximum	Coded Low	Coded High	Mean	Std. Dev.
A	L/P ratio	w/w	Numeric	Continuous	0.7929	2.21	-1 ↔ 1.00	+1 ↔ 2.00	1.50	0.4714
B	A/O ratio	v/v	Numeric	Continuous	3.17	8.83	-1 ↔ 4.00	+1 ↔ 8.00	6.00	1.89

Table S12. ANOVA for Quadratic model of LPHNs' particle size response

Source	Sum of Squares	df	Mean Square	F-value	p-value	
Block	2153.97	1	2153.97			
**Model**	13417.24	5	2683.45	104.94	0.0095	significant
A-L/P ratio	4771.13	1	4771.13	186.58	0.0053	
B-A/O ratio	7603.94	1	7603.94	297.37	0.0033	
AB	408.71	1	408.71	15.98	0.0572	
A^2	51.05	1	51.05	2.00	0.2932	
B^2	1032.03	1	1032.03	40.36	0.0239	
**Residual**	51.14	2	25.57			
**Cor Total**	15622.34	8				

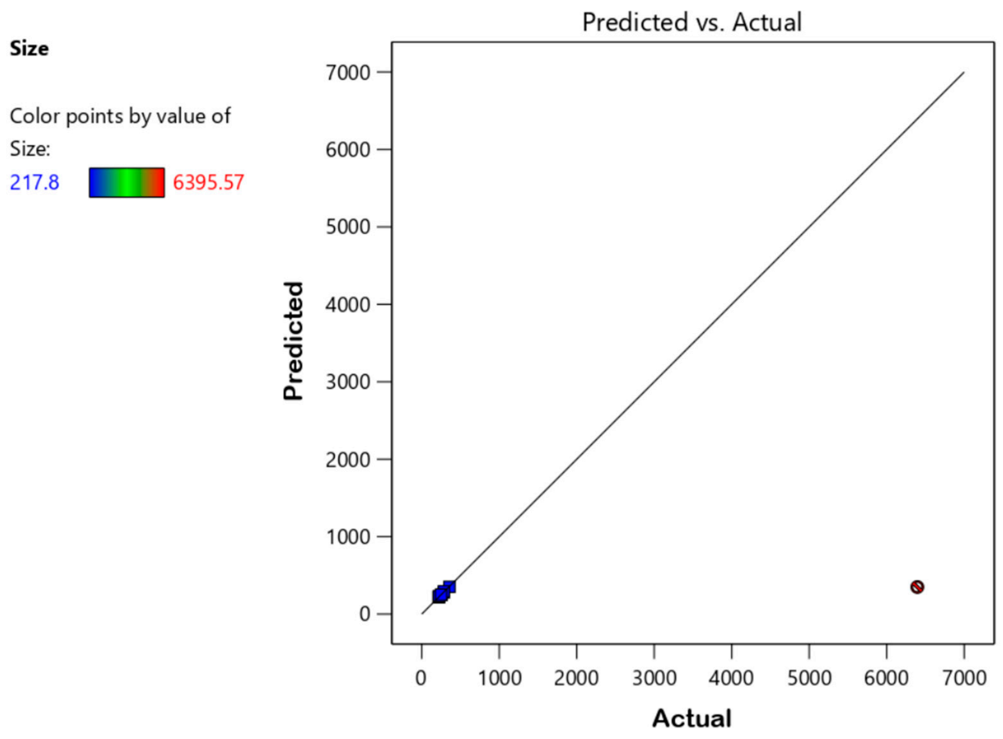

Figure S6. Scatter plot of predicted versus observed data for particle size response of LPHNs.

Table S13. ANOVA for Quadratic model of LPHNs' PDI response

Source	Sum of Squares	df	Mean Square	F-value	p-value	
Block	0.0080	1	0.0080			
**Model**	0.0333	5	0.0067	58.03	0.0170	significant
A-L/P ratio	0.0002	1	0.0002	1.35	0.3650	
B-A/O ratio	0.0293	1	0.0293	255.34	0.0039	
AB	0.0002	1	0.0002	1.67	0.3258	
A^2	0.0002	1	0.0002	1.73	0.3194	
B^2	0.0022	1	0.0022	19.05	0.0487	
**Residual**	0.0002	2	0.0001			
**Cor Total**	0.0415	8				

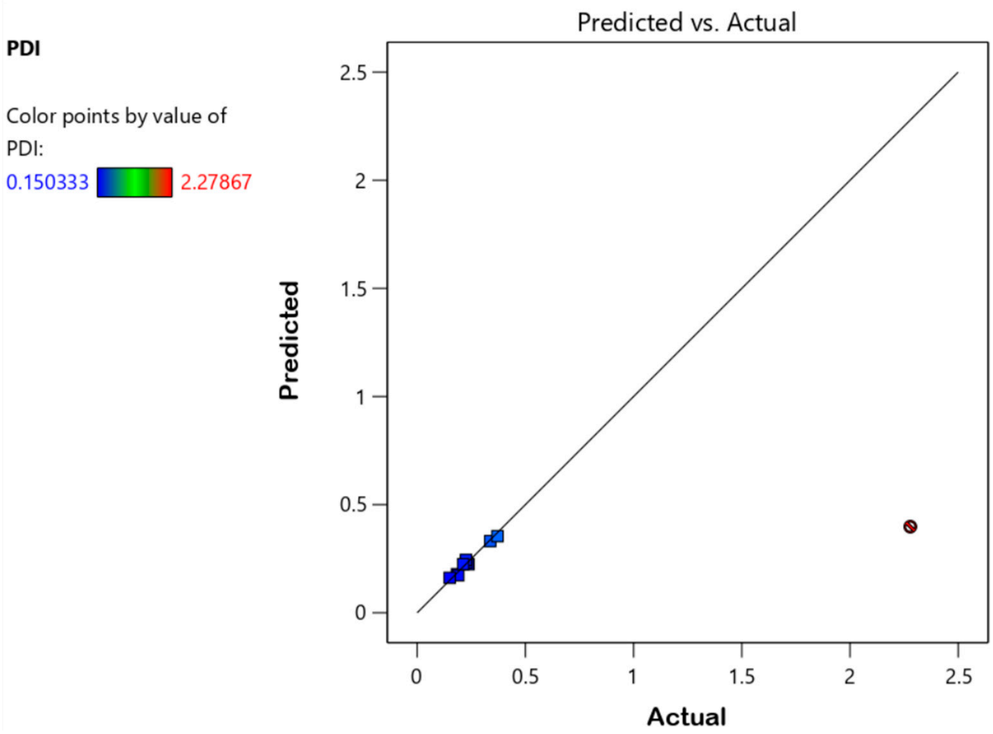

Figure S7. Scatter plot of predicted versus observed data for PDI response of LPHNs.

Table S14. Logistic Regression (Type III) for formulation failure response of LPHNs

**Source**	**df**	χ^2	**p-value**
Block	1		
**Model**	1	6.50	0.0108
B-A/O ratio	1	5.00	0.0253

χ^2 Log Likelihood Ratio p-values

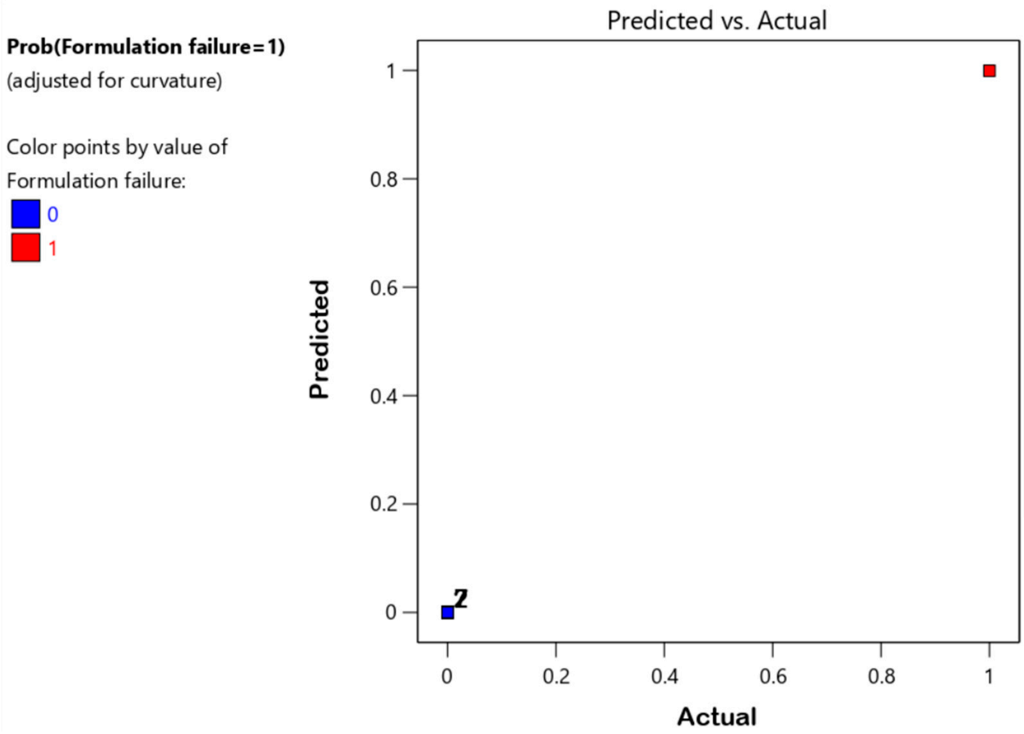

Figure S8. Scatter plot of predicted versus observed data for formulation failure response of LPHNs.

Table S15. Fit Summary for ZP response of LPHNs

Source	Sequential p-value	Lack of Fit p-value	Adjusted R^2	Predicted R^2	
**Linear**	**0.1442**		**0.3009**	**-0.4749**	**Suggested**
2FI	0.4739		0.2509	-1.3806	
Quadratic	0.7077		0.0084	-3.4024	
Cubic	0.4980		0.2622	-28.9749	**Aliased**

Table S16. ANOVA for Linear model of LPHNs' ZP response

Source	Sum of Squares	df	Mean Square	F-value	p-value	
Block	40.00	1	40.00			
**Model**	29.01	2	14.51	2.72	0.1442	not significant
A-L/P ratio	27.12	1	27.12	5.09	0.0649	
B-A/O ratio	1.89	1	1.89	0.3545	0.5733	
**Residual**	31.98	6	5.33			
**Cor Total**	100.99	9				

Table S17. Fit statistics for ZP response of LPHNs

**Std. Dev.**	2.31	**R^2**	0.4757
**Mean**	-43.44	**Adjusted R^2**	0.3009
**C.V. %**	5.31	**Predicted R^2**	-0.4749
		**Adeq Precision**	6.1168

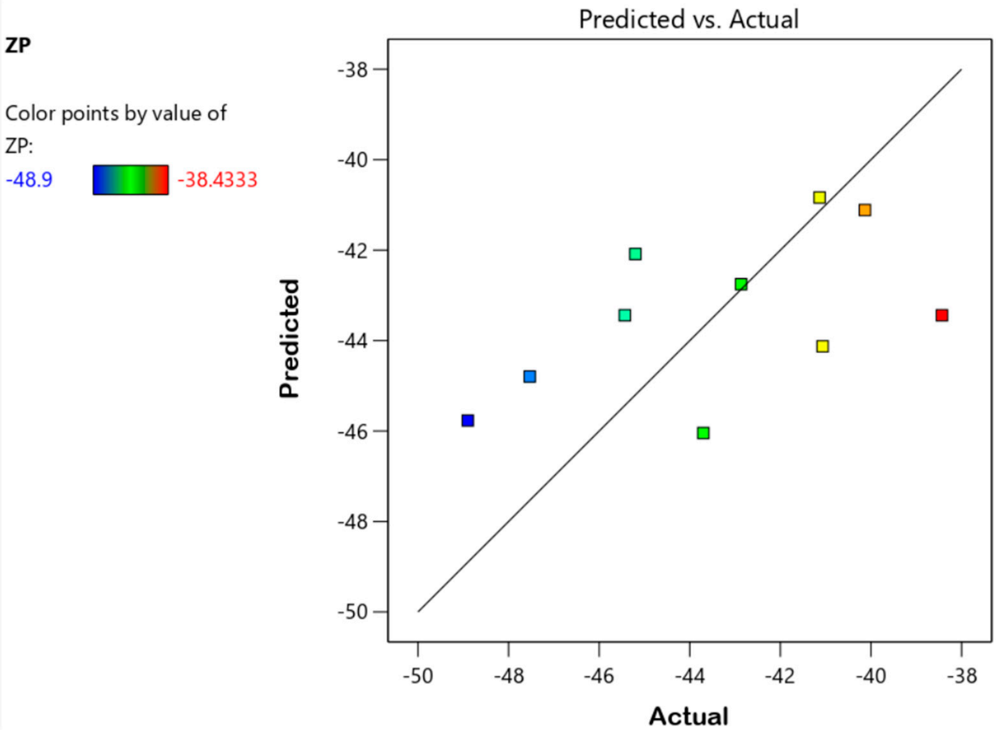

Figure S9. Scatter plot of predicted versus observed data for ZP response of LPHNs.

Table S18. ANOVA for 2FI model of EE response of LPHNs

Source	Sum of Squares	df	Mean Square	F-value	p-value	
Block	2.34	1	2.34			
**Model**	804.15	3	268.05	37.59	0.0007	significant
A-L/P ratio	651.01	1	651.01	91.29	0.0002	
B-A/O ratio	126.29	1	126.29	17.71	0.0084	
AB	26.85	1	26.85	3.77	0.1100	
**Residual**	35.66	5	7.13			
**Cor Total**	842.14	9				

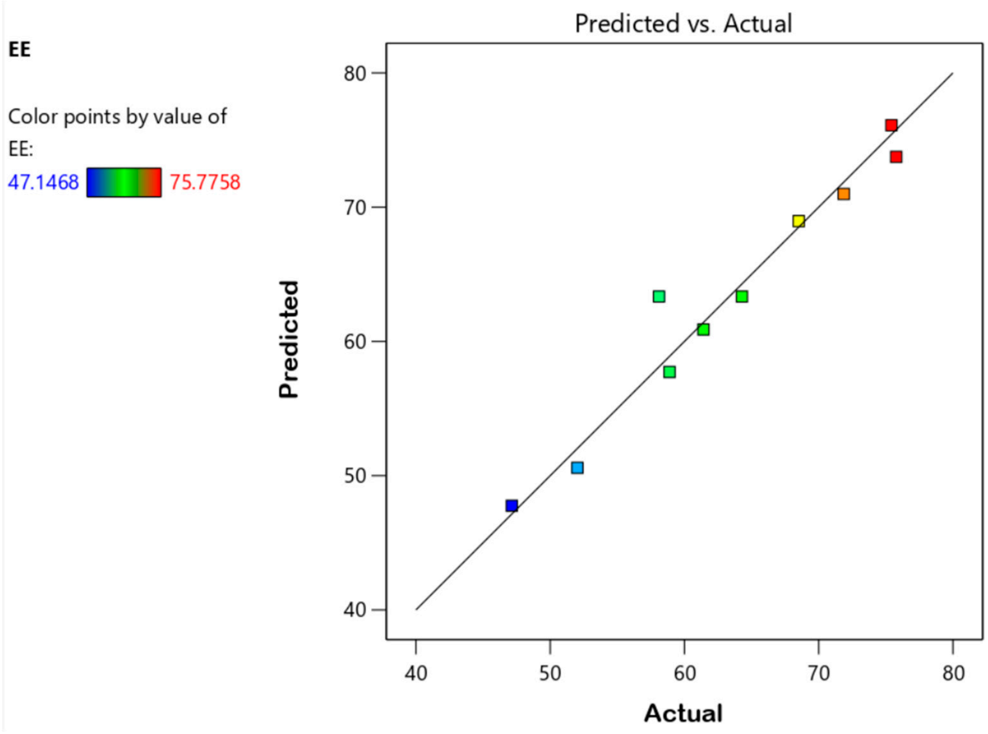

Figure S10. Scatter plot of predicted versus observed data for EE response of LPHNs.

Table S19. ANOVA for Quadratic model of DLE response of LPHNs

Source	Sum of Squares	df	Mean Square	F-value	p-value	
Block	0.1403	1	0.1403			
**Model**	262.77	5	52.55	133.28	0.0010	significant
A-L/P ratio	248.67	1	248.67	630.63	0.0001	
B-A/O ratio	6.74	1	6.74	17.10	0.0257	
AB	1.13	1	1.13	2.87	0.1890	
A^2	6.04	1	6.04	15.33	0.0296	
B^2	0.4503	1	0.4503	1.14	0.3636	
**Residual**	1.18	3	0.3943			
**Cor Total**	264.09	9				

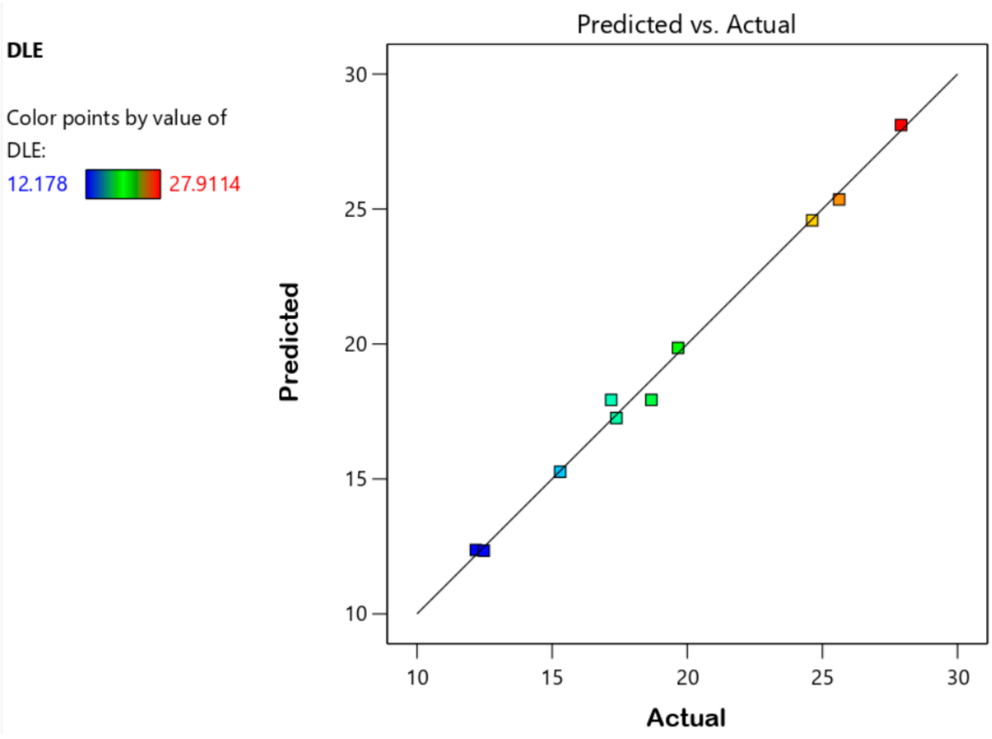

Figure S11. Scatter plot of predicted versus observed data for DLE response of LPHNs.

Table S20. Constraints used for optimization of LPHNs

Name	Goal	Lower Limit	Upper Limit	Lower Weight	Upper Weight	Importance
A:L/P ratio	is in range	1	2	1	1	3
B:A/O ratio	is in range	4	8	1	1	3
Size	minimize	217.8	357.267	1	1	3
PDI	minimize	0.150333	0.371333	1	1	3
Prob(Formulation failure=1)	minimize	0.001	0.999	1	1	3
ZP	none	-48.9	-38.4333	1	1	3
EE	maximize	47.1468	75.7758	1	1	3
DLE	maximize	12.178	27.9114	1	1	3

Table S21. LPHN optimization solutions suggested by the software based on pre-defined criteria

Number	L/P ratio	A/O ratio	Size	PDI	Prob(Formulation failure=1)	ZP	EE	DLE	Desirability	
**1**	**1.000**	**8.000**	**245.676**	**0.178**	**0.000**	**-41.113**	**70.985**	**24.5815**	**0.856**	**Selected**

Table S22. Coefficients Table summarizing the effects of different factors on the studied responses of LPHNs

	Intercept	Block [1]	A	B	AB	A^2	B^2
**Size**	251.6	5.54429	**-24.4211**	**-42.5979**	10.1083	3.44852	**18.9128**
**p-values**			**0.0053**	**0.0033**	0.0572	0.2932	**0.0239**
**PDI**	0.225	0.0117381	0.0044036	**-0.0836448**	-0.00691667	0.00679465	**0.0275327**
**p-values**			0.3650	**0.0039**	0.3258	0.3194	**0.0487**
**Logit[Prob(Formulation failure=1)]**	-45.8424	-18.8932		-37.6033			
**p-values**							
**ZP**	-43.44	-2	-1.84123	0.485969			
**p-values**			0.0649	0.5733			
**EE**	63.3462	-0.483337	**-9.02087**	**-3.97319**	-2.591		
**p-values**			**0.0002**	**0.0084**	0.1100		
**DLE**	17.9289	-0.118461	**-5.57533**	**-0.917975**	-0.531631	**1.1498**	0.313856
**p-values**			**0.0001**	**0.0257**	0.1890	**0.0296**	0.3636

p-value shading: **p < 0.05** 0.05 ≤ p < 0.1 p ≥ 0.1
